# A dynamical view of protein-protein complexes: Studies by molecular dynamics simulations

**DOI:** 10.3389/fmolb.2022.970109

**Published:** 2022-10-06

**Authors:** Juliette Martin, Elisa Frezza

**Affiliations:** ^1^ Univ Lyon, Université Claude Bernard Lyon 1, CNRS, UMR 5086 MMSB, Lyon, France; ^2^ Université Paris Cité, CiTCoM, Paris, France

**Keywords:** protein-protein interactions (PPI), protein flexibility, protein-protein interface analysis, structural water molecule, molecular dynamics simulation, flexibility of protein-protein complexes

## Abstract

Protein-protein interactions are at the basis of many protein functions, and the knowledge of 3D structures of protein-protein complexes provides structural, mechanical and dynamical pieces of information essential to understand these functions. Protein-protein interfaces can be seen as stable, organized regions where residues from different partners form non-covalent interactions that are responsible for interaction specificity and strength. They are commonly described as a peripheral region, whose role is to protect the core region that concentrates the most contributing interactions, from the solvent. To get insights into the dynamics of protein-protein complexes, we carried out all-atom molecular dynamics simulations in explicit solvent on eight different protein-protein complexes of different functional class and interface size by taking into account the bound and unbound forms. On the one hand, we characterized structural changes upon binding of the proteins, and on the other hand we extensively analyzed the interfaces and the structural waters involved in the binding. Based on our analysis, in 6 cases out of 8, the interfaces rearranged during the simulation time, in stable and long-lived substates with alternative residue-residue contacts. These rearrangements are not restricted to side-chain fluctuations in the periphery but also affect the core interface. Finally, the analysis of the waters at the interface and involved in the binding pointed out the importance to take into account their role in the estimation of the interaction strength.

## 1 Introduction

Protein-protein interactions play an essential role in the biological function of many proteins, including gene expression, metabolism, regulation and transport (Jones and Thornton, 1996; Alberts, 1998; Minton, 2000; Ellis and Minton, 2003; Perkins et al., 2010). However, single point mutation can perturb these interactions leading to several diseases (Stites, 1997; Schuster-Böckler and Bateman, 2008; Cafarelli et al., 2017). Therefore, characterizing these interactions properly and in particular protein-protein interfaces is a crucial step in gaining an understanding of mechanisms of biological processes in cells (Zhang et al., 2016). Protein-protein interfaces also constitute a valid target for drug discovery *via* the identification of the hotspots (Shin et al., 2020). In this context, the knowledge of the 3D structure of the complexes is fundamental to explore the recognition processes at atomic level and to properly characterize their interactions.

Our current understanding of protein-protein interfaces has grown along with the amount of structural information deposited in the Protein Data Bank (PDB) (Berman et al., 2000). Protein-protein interfaces span a large range of size and shape, display shape complementarity, and are usually described in terms of core and peripheral regions, with a few hotspot residues being the major contributors to the binding affinity (Clackson and Wells, 1995; Jones and Thornton, 1996; Bogan and Thorn, 1998; Chakrabarti and Janin, 2002; Saha et al., 2006; Levy, 2010). However, the anatomic description of protein-protein interfaces and their physico-chemical properties generally relies on a static point of view. In the literature, only some complexes have also been studied *via* Molecular Dynamics (MD) simulations, in order to probe their association mechanism (Ahmad et al., 2011, Ahmad et al.,2008; Barducci et al., 2013; Schmidt et al., 2013; Abriata and Peraro, 2015; Blöchliger et al., 2015; Fuchs et al., 2015; Paul et al., 2017; Plattner et al., 2017; Saglam et al., 2017; Pan et al., 2019). Concerning the interfaces, Shaw and co-workers performed hundred-microsecond MD simulations of the protein-protein association in five complexes which allowed observing transition states before native association, with low fraction of native contacts and highly hydrated interfaces (Pan et al., 2019). Two recent studies of models obtained by protein-protein docking have shown that their interfaces undergo significant changes in simulations, and also observed changes in the interfaces of native complexes (Jandova et al., 2021; Prévost and Sacquin-Mora, 2021). Similar findings were also obtained by us for protein-DNA complexes. First, some of us observed that some protein-DNA interfaces display distinct conformational substates that recognize different parts of the consensus DNA sequences (Etheve et al., 2016a; Etheve et al., 2016b). Secondly, in another study, it has been shown that the stability of the protein-DNA interfaces depends on the specificity of the interactions, in particular a non-specific DNA sequence can investigate several distinct relative conformations with respect to the protein and at the same time the strength of the complex is only partially affected (Carzaniga et al., 2021).

Another key aspect to discriminate between interacting or non-interacting proteins and to quantify their interaction is the binding affinity. Commonly, the strength of the interaction is defined through the equilibrium dissociation constant *K_d_* or the Gibbs free energy difference *(∆G_b_ = -RT ln K_d_)* (Kastritis and Bonvin, 2013a, Kastritis and Bonvin, 2013b). Experimentally, several methods with different sensitivity and accuracy can be exploited to determine it (Piehler, 2005; Shoemaker and Panchenko, 2007; Liao et al., 2021). From the computational point of view, several methods have been developed with different computational costs. On the one hand, for example, alchemical calculations are quite accurate, but they are still very challenging for protein-protein complexes (Gapsys et al., 2016; Mey et al., 2020; Siebenmorgen and Zacharias, 2020). On the other hand, faster and less accurate approaches, such as Molecular Mechanics Poisson-Boltzmann Surface Area (MM-PBSA) methods (Gohlke and Klebe, 2002; Hou et al., 2011; Wang et al., 2018), are widely used to predict the binding free energy. In this context, the Gibbs free energy difference is decomposed into an enthalpic and an entropic contribution. The former is the difference between bonded and non-bonded interactions between interacting partners, which is partially related to atomic interactions at the interface, among them for example hydrogen bonds and hydrophobic contacts. The latter accounts for the loss/gain of entropy of the partners upon complex formation and the entropic gain of water molecules released from the protein interface. For the enthalpic contribution, it is usual to sample the conformational landscape of the complex by MD (Hou et al., 2011). This, in principle, should help to better approximate the average enthalpic term, by neglecting local/short-time fluctuations. The entropic contribution is notably difficult to estimate, in particular when Cartesian coordinates are used, since it can require long MD simulations to reach convergence and its difference can be affected by this instability. To improve this, several groups proposed to compute the conformational entropy using internal variables (Harpole and Sharp, 2011), such as torsions (Chong and Ham, 2016b; Hikiri et al., 2016; Wickstrom et al., 2022). However, the prediction of the binding free energy based purely on physical contributions remains challenging due to the approximations used and the computational time required with more advanced methods.

In this context, water molecules also play a crucial role in the process of binding, in specific recognition and in protein-protein interactions (Laage et al., 2017). In fact, they seem to be involved in mediating interactions (Papoian et al., 2003), like in the approach of one protein to another as observed by the formation of an adhesive hydrogen-bond network between the interfaces stabilizing early intermediates before native contacts are formed (Ahmad et al., 2011). At the interface, water molecules play a key structural role in determining the stability and specificity of biomolecular assembly. Moreover, the dynamics of water molecules slows down at the protein-protein interface due to confinement effects (Chong and Ham, 2016a) as also observed in cavities (Macro et al., 2021). Although several PDB structures have been analyzed (Rodier et al., 2005), a static view has often been presented and only few studies have been conducted that take into account the interplay between water and protein(s) dynamics (Huggins et al., 2011). In the characterization and determination of protein-protein interactions, the role of water molecules is also related to the solvation entropy that still has to be deeply understood and taken into account properly. The crucial role of water in protein-protein interactions has also been supported by the fact that taking into account explicit interface water molecules could improve the performance of protein-protein docking (van Dijk and Bonvin, 2006; Parikh and Kellogg, 2013).

In this study, we aim to continue this effort to characterize the dynamics of protein-protein complexes and their interfaces at molecular level. To get insights on the dynamics of protein-protein complexes, we study a set of eight transient protein-protein complexes for which high resolution crystallographic 3D structures are available and thermodynamic parameters are reported in the Affinity Benchmark (Kastritis et al., 2011). We performed all-atom Molecular Dynamics simulations of complexes and free unbound proteins in explicit water at physiological conditions. MD simulations were extensively characterized and several analyses were carried out. Among them, we focused on the characterization of the stability of their interfaces and hydration shells. In each system, we analyzed in detail the contacts between interface residues over time and the presence of water at the interfaces.

## 2 Materials and methods

### 2.1 Data set

**FIGURE 1:**
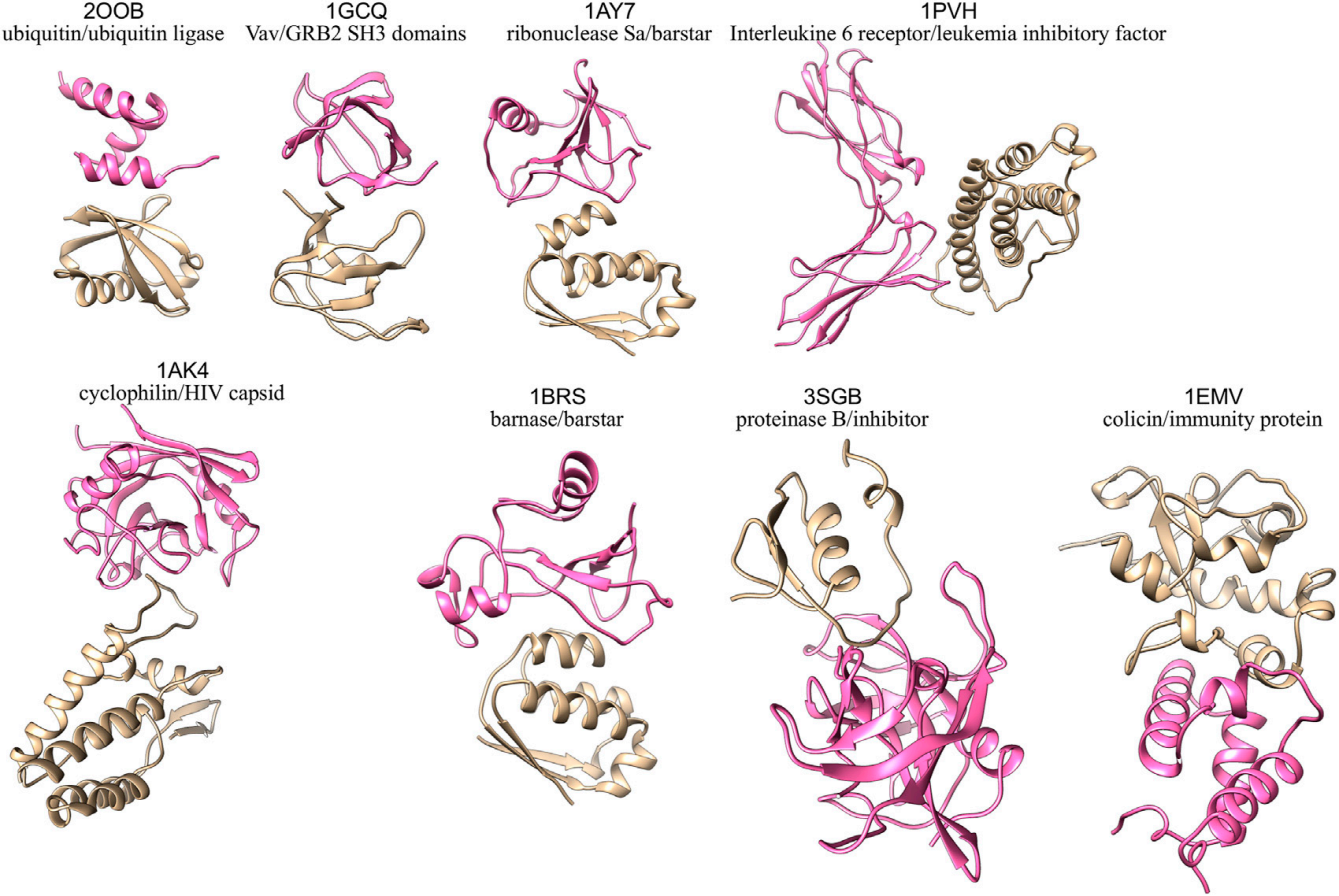
3D structures of the eight protein-protein complexes simulated in this study, with one partner colored in pink and the other one in beige.

Eight binary protein-protein complexes from the Docking benchmark (Hwang et al., 2010) and the Affinity benchmark (Kastritis et al., 2011) are studied in the present work (see Supplementary Table S1). The 3D structures of the eight complexes under study are shown in Figure 1. To facilitate subsequent analyses, we chose exclusively rigid-body cases, i.e., with limited conformational changes at the interface between the bound and unbound forms. They are classified in different functional classes, with a predominance of enzyme-containing complexes, reflecting the structural data available: enzyme/substrate (ubiquitin/ubiquitin ligase complex 2OOB (Peschard et al., 2007)), enzyme/inhibitor (ribonuclease Sa/barstar complex 1AY7 (Sevcík et al., 1998), barnase/barstar complex 1BRS (Buckle et al., 1994), proteinase B/inhibitor 3SGB (Read et al., 1983) and colicin/immunity protein complex 1EMV (Kühlmann et al., 2000)), receptor containing complex (complex between Interleukine six receptor and leukemia inhibitory factor, code 1PVH (Boulanger et al., 2003)) and “other” (Vav/GRB2 SH3 domains complex 1GCQ (Nishida et al., 2001) and cyclophilin/HIV capsid complex 1AK4 (Gamble et al., 1996)). The interface size and binding affinity of each complex are given in Supplementary Table S1. As can be seen in Supplementary Table S1, the eight cases span various interface sizes (700–1,400 Å^2^) and experimental binding affinities (−5.7 to −18.6 kcal/mol). To model our starting structures, we verified that there were no mutations in the unbound form with respect to the bound complex, no gap in the backbone and we used the same number of residues for the complex and the free proteins. Terminal residues present in the free proteins but absent from the complex were chopped, and residues present in the complex but absent in the free proteins were modeled based on the complex. We computed a pKa calculation on the complex structure using the software PDB2PQR (Dolinsky et al., 2004) and the algorithm PROPKA (Olsson et al., 2011; Sondergaard et al., 2011) to define the protonation state of each residue. To deal with the same chemical species, we applied the same protonation state of the complex on the relative free proteins.

### 2.2 All-atom molecular dynamics simulations

All-atom Molecular dynamics (MD) simulations were performed with the GROMACS five package (Berendsen et al., 1995; Lindahl et al., 2001; Van Der Spoel et al., 2005; Hess et al., 2008; Abraham et al., 2015) using the Amber 99SB-ILDN force field for proteins (Wang et al., 2000; Hornak et al., 2006; Lindorff-Larsen et al., 2010). The proteins we studied were each placed in a truncated octahedral box and solvated with TIP3P water molecules (Jorgensen et al., 1983) to a depth of at least 11 Å. The solute was neutralized with potassium cations and then K^+^Cl^−^ ion pairs (Dang, 1995) were added to reach a physiological salt concentration of 0.15 M. Long-range electrostatic interactions were treated using the particle mesh Ewald method (Darden et al., 1993; Essmann et al., 1995) with a real-space cutoff of 10 Å. Bond lengths were restrained using P-LINCS (Hess et al., 1997), allowing a time step of 2 fs (Berendsen et al., 1984b). Translational movement of the solute was removed every 1,000 steps to avoid any kinetic energy build-up (Harvey et al., 1998). After energy minimization of the solvent and equilibration of the solvated system for 10 ns using a Berendsen thermostat (t_T_ = 1 ps) and Berendsen pressure coupling (t_P_ = 4 ps) (Berendsen et al., 1984a) by slowing relaxing the position restraints on the backbone, the simulations were carried out in an NTP ensemble at a temperature of 300 K and a pressure of 1 bar using a Bussi velocity-rescaling thermostat (Bussi et al., 2007) (*τ*_T_ = 1 ps) and a Parrinello-Rahman barostat (*τ*_P_ = 1 ps) (Parrinello and Rahman, 1981) for 10 ns before starting the production run. Simulations were carried out using typically between 72 and 120 computer cores depending on the system size, which allowed a production rate of about 100 ns/day. Each simulation was 300 ns long, except for the larger complexes 1PVH (complex between Interleukine six receptor and leukemia inhibitory factor) and 1AK4 (cyclophilin/HIV capsin complex) which were simulated for 500 ns. A total simulation time of 8.4 μs was performed.

### 2.3 RMSD/RMSF analysis of complexes and single proteins

We computed the clustering analysis based on backbone RMSD on the complexes and the single proteins using the algorithm TTClust (Tubiana et al., 2018). For the complex, we computed the clustering analysis using a 2 Å cutoff on the whole complex and on the separate proteins. To understand the impact of the binding, we computed the RMSD time series on backbone atoms separately on each protein for the complexes and we used either the starting structure or the central structure of the largest cluster obtained for the single protein simulation as reference.

For RMSF calculations, we first superimposed the trajectory on the starting structure for the unbound proteins and on the separated chains for complexes. We computed RMSF only for the backbone atoms and we averaged the fluctuations for each residue. This procedure allowed us to study the change of flexibility upon binding for each chain.

### 2.4 Interface analysis

The interface analysis is conducted on MD snapshots taken every ns (i.e., 300 to 500 snapshots depending on the system). Interface RMSD (iRMSD) between different snapshot was computed with DockQ (Basu and Wallner, 2016).

#### 2.4.1 Interface definition

Interface contacts are defined at the residue level using a 5 Å distance cutoff between heavy atoms. The solvent accessible surface area (ASA) is computed with the NACCESS software using the default radius of 1.4 Å to take into account the water molecule (Hubbard and Thornton, 1992)**.**

We define distinct regions at the interface according to the criteria introduced by Levy (Levy, 2010). Residues with a change of ASA between the isolated chain and the complex are classified as: support if the relative ASA in the isolated chain extracted from the complex is lower than 25%, *rim* if relative ASA in the complex is greater than 25%, *core* if relative ASA in the isolated chain is greater than 25% and lower than 25% in the complex.

#### 2.4.2 Interface properties

The area of accessible surface buried by the interface is defined by:

$$\Delta ASA=ASA_{A}+ASA_{B}-ASA_{AB}$$ (1)

where ASA_A_ and ASA_B_ denote the accessible surface areas of separate chains and ASA_AB_ the accessible surface area of the complex.

The gap index is defined as

gap index=2 gap volume / ΔASA (2)

where the gap volume is computed with the SURFNET software (Laskowski, 1995).

The number of hydrogen bonds at the interface is detected using the *gmx hbond* routine implemented in GROMACS (Abraham et al., 2015).

#### 2.4.3 Interface clustering

To compare the interfaces at different points in time, the two corresponding snapshots S1 and S2 are described in terms of their interface contacts. The interface similarity is then measured by the Jaccard index *J* between the two sets of contacts C1 and C2, defined as:

$$J\left(C1,C2\right)=\frac{\left|C1\cap C2\right|}{\left|C1\cup C2\right|}$$ (3)

The Jaccard index is equal to 0 if S1 and S2 have no common contacts; it is equal to one if S1 and S2 have identical contacts.

The Jaccard indexes are transformed into dissimilarity matrices by taking 1-*J*, and hierarchical clustering is applied to identify clusters at the interfaces. We used the Ward. D2 method implemented in R (Murtagh and Legendre, 2014). There is no generic method to choose the optimal number of clusters, which depends on the application. In our case, the goal is to have clusters that are different enough from each other, of reasonable size, and relatively stable in time. The choice of the optimal number of clusters was thus guided by the topology of the clustering dendrograms and the assessment of cluster size and stability in time when varying the number of clusters. We thus computed the size of the smallest cluster (number of snapshots in this cluster), the size of the largest cluster, and the number of cluster changes during the simulation time.

#### 2.4.4 Choice of representative structures in each cluster

In each cluster, we defined the centroid as the snapshot with the highest average Jaccard similarity with the other snapshots of the cluster. To visualize the location of these centroids in each cluster, we applied principal component analysis on the Jaccard dissimilarity matrices and plotted the first two dimensions. Projections are shown in Supplementary Figure S1.

#### 2.4.5 Statistics of interface contacts in the different clusters

We computed the relative frequency of interface contacts in each cluster, defined by the proportion of snapshots exhibiting a contact in the cluster. Each contact is thus associated with Nc values of relative frequencies, where Nc is the number of clusters. For each contact, we computed the variance of these values across clusters. This indicates which contacts have variable frequencies across clusters (high variance), and which have similar frequencies (low variance).

Variance values refer to contacts between residue pairs. To map these variance values at the level of residues, we selected, for each residue, the maximum variance observed across contacts:

$$var\;\left(X\right)=\max_{\;Y\;in\;contacting\;residues\;}\left\{\;var\left(XY\right)\right\}$$ (4)

where var (*XY*) is the variance of the relative frequency of the *XY* contact across clusters. For example, a residue *X* seen in contact with residue *Y* with a variance of 0.1 and in with residue *Z* with a variance of 0.3 will receive a value of 0.3.

We also derived a recurrence index, to quantify the recurrence of each residue in contacts across clusters. It is defined by:

$$recurrence\;index\;\left(X\right)=\min_{c\;in\;clusters}\left\{\max_{Y\;in\;contacting\;residues}\left\{Fc\left(XY\right)\right\}\;\right\}$$ (5)

where Fc(*XY*) is the relative frequency of contact between *X* and *Y*. The inner maximum is taken over interacting residues in each cluster, and the outer minimum is taken over clusters. For example, if a residue *X* is seen at 100% in contact with residue *Y* and 4% with residue *Z* in one cluster, and 10% in contact with residue *Y* and 60% with residue *W*, its recurrence index is equal to 60%. This allows discriminating between residues that are part of the interface in every cluster (high recurrence index) and those that come and go (low recurrence index), without being affected by the unbalance between clusters.

### 2.5 Mutation data

Free energy changes associated with mutations in the complexes under study were extracted from SKEMPI 2.0 (Jankauskaitė et al., 2019). Multiple mutations are not taken into account. Hotspots are defined as residues with ΔΔG < −2.0 kcal/mol.

### 2.6 Water analysis

#### 2.6.1 Nonpolar solvation dispersion energy and release of water molecules

In order to compute the nonpolar solvation dispersion energy (∆*G*_np,disp, solv_) between the protein and the water molecules, we took *N* snapshots from the MD simulations separated by 1 ns, we minimized them by turning off all electrostatics interactions and we ran 50 ps using the ensemble *NVT*. Then, we determined the dispersion energy between the protein(s) and the water for each snapshot and we averaged it and used it as the repulsive part of the nonpolar solvation energy for the macromolecule.

Moreover, we computed the release of water molecules upon complexation, which can be related to another term of nonpolar solvation energy. To do so, we need to define a hydration shell, which is defined to include all the water molecules whose distance from the protein is beyond a certain cutoff distance. We computed the number of water molecules as a function of the minimal distance (*d*_*m*_) of the water O and the heavy atoms of the protein (see Supplementary Figure S2). To do so, we considered an interval of 0.1 Å for each bin and we determined the number of water molecules whose distance *d*_*m*_ from the protein is within the given interval. The resulting criteria are R_min,1_ < 3.4 Å for the first shell and 3.4 ≤ R_min,2_ < 5.0 Å for the second shell.

#### 2.6.2 Interfacial water molecules

To study the water molecules at the protein-protein interface, we determine the water molecules whose distance from both proteins is below 4 Å. To compute this distance, we only considered the heavy atoms of protein(s) and the oxygen of the water molecules as for determining the number of water molecules released. In this manuscript we referred to these water molecules as interfacial water molecules. Interfacial water molecules were determined for the representative structures and along MD simulations. For the latter, we also analyzed the contacts formed for each interfacial water molecule with the same distance cutoff and the two protein partners to investigate the contacts mediated by water.

### 2.7 Statistical testing

The correlation between variables was assessed using the Pearson correlation coefficient. The difference between two correlation coefficients was assessed using the Dunn and Clark test (Dunn and Clark, 1969) implemented in the R package cocor (Diedenhofen and Musch, 2015), that takes into account the intercorrelation between variables in the case of dependent groups.

## 3 Results

### 3.1 Global conformational changes observed in molecular dynamics

#### 3.1.1 RMSD clustering and RMSF profiles of complexes and single proteins

As presented in the Section Material and Methods, we performed clustering analysis based on backbone RMSD on the complexes and the single proteins and we computed the RMSD time series. RMSD time series and clustering results are summarized in Supplementary Figures S3–S5. When simulated in the free state, all the proteins under study display either low (e.g., 1BRS) or moderate conformational changes, usually confined to tails (e.g., 2OOB, 1GCQ, and 1PVH) or some particular loops (e.g., 1AY7, 1AK4, 3SBG, and 1EMV). RMSF profiles are shown in Supplementary Figure S6, with 3D structures colored according to the change of flexibility between free states and complexes. Based on these profiles, the flexibility of proteins in complexes are usually comparable to what is seen in the unbound form. However, we also observed that the formation of the complex can induce increased flexibility at sites distant from the interface due to allosteric communication. Two complexes show a significant reorientation of the two proteins during the simulation time: the cyclophilin/HIV capsid complex 1AK4 (Supplementary Figure S5A), and, to a lesser extent, the proteinase B/inhibitor complex 3SGB (Supplementary Figure S5C).

Interestingly, proteins interact in a similar way in these two complexes: in 1AK4, a loop of the HIV capsid is inserted in the cyclophilin binding site and in 3SGB, a loop of the inhibitor is inserted in the active site of the protease (see Figure 1). In both cases, the loops that are responsible for the interactions are flexible (see Supplementary Figures S3, S5, S6). This flexibility, coupled to the binding site topology, results in a pivotal movement of one protein with respect to the other reflected by a high global RMSD. Thus, even with rigid body proteins, we observe different dynamic behaviors, resulting from the intrinsic flexibility of the proteins and the topology of their binding sites. We can speculate that these different dynamic behaviors may be related to different biological functions.

### 3.2 Geometric interface parameters

**FIGURE 2:**
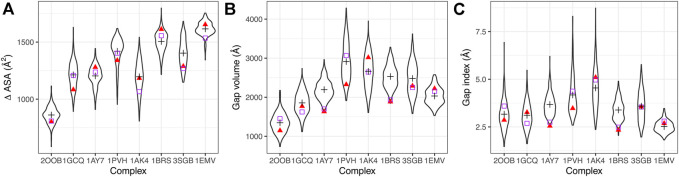
Geometric interface parameters. **(A)** Interface size measured by ∆ASA, **(B)** gap volume, **(C)** gap index. Distributions of values sampled during the simulations are represented in black as violin plots, with the average value indicated by a black cross. Red triangles indicate the initial values (t = 0), purple squares the values in the X-ray structures.

To understand the impact of the dynamics on the geometric parameters, we computed the ∆ASA, the gap volume and the gap index on the X-ray complexes and along MD trajectories. Distributions are shown in Figure 2 and average values in Supplementary Table S2.

We compared the interface properties between the starting X-ray structures, the equilibrated structures at the beginning of the production time (*t* = 0) and the average value obtained along the trajectory. All geometric interface parameters show variability during the simulation. Even though the initial values can differ from the crystallographic ones, the simulations sample values around the crystallographic one in most of the cases (see for example complex 1GCQ in Figure 2A). In some cases, however, we observe a significant change: the interfaces in complexes 1AK4 and 3SGB become larger (see Figure 2A); the interfaces in complexes 1AY7 and 1BRS increase in terms of gap volume (see Figure 2B) and gap index (see Figure 2C). Interestingly, we also observed that the variability of the gap index is related to the strength of interaction: large variability corresponds to lower interaction strength (see Supplementary Figure S7). This highlights the need to characterize the role of dynamics of the interface in the binding affinity.

### 3.3 Dynamics of interfaces

#### 3.3.1 Conservation of initial interface contacts

**FIGURE 3:**
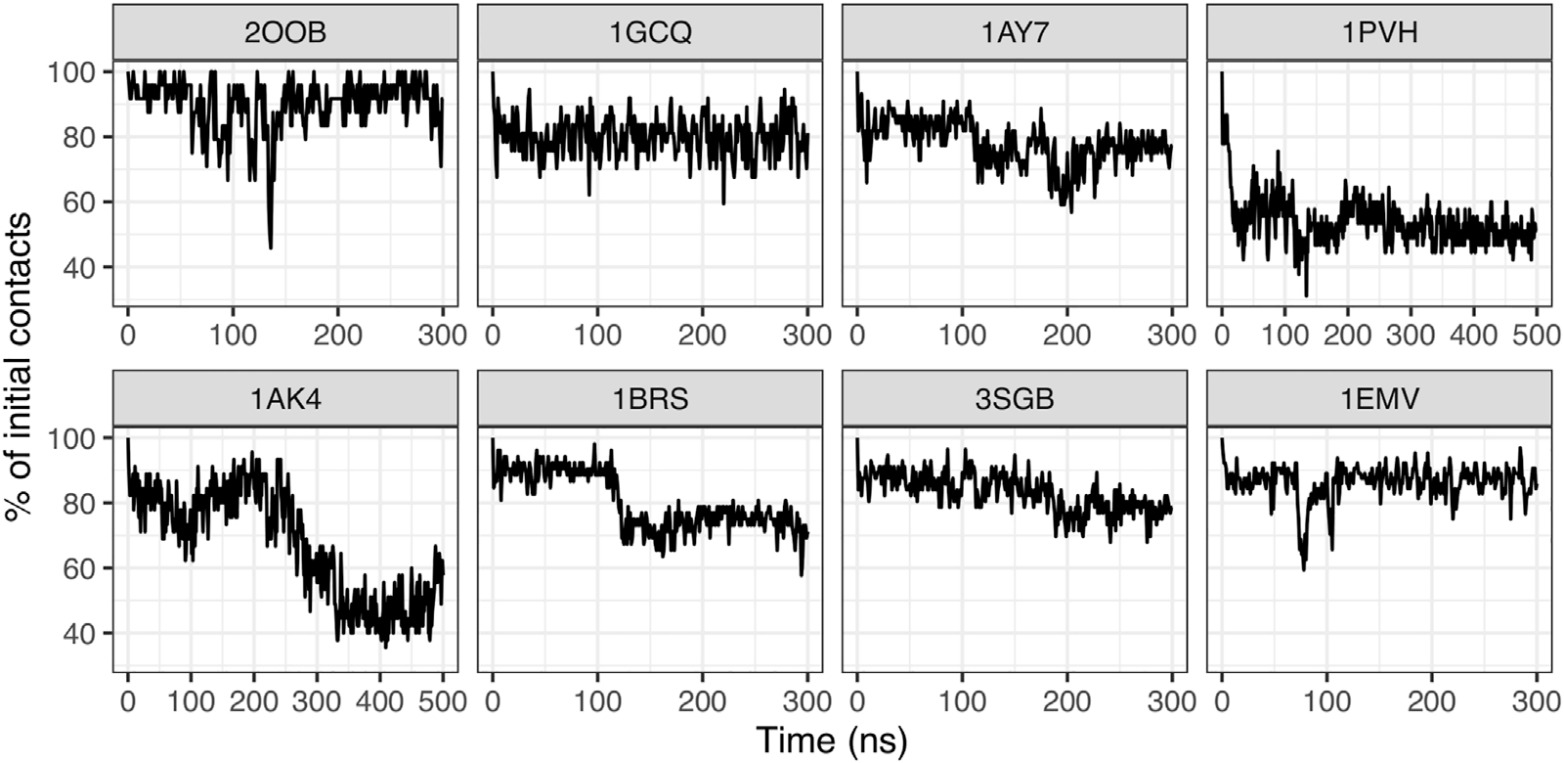
Time series of the conservation of initial contacts.

We computed interface contacts along the MD trajectories in each complex, as explained in the Material and Method section. Interface contacts are computed for each snapshot taken at every 1 ns; we then compute the fraction of initial contacts that is preserved during simulation time. As shown in Figure 3, the stability of contacts is variable across different protein-protein complexes. The ubiquitin/ubiquitin ligase complex 2OOB, which has the smallest interface in our data set (ΔASA = 808 Å^2^) has a very stable interface, with about 90% of initial contacts preserved during the 300 ns of simulation. The SH3 domain complex 1GCQ (medium size interface, ΔASA = 1,207 Å^2^) also has a very stable interface, with about 80% of initial contacts preserved. In all other complexes (ΔASA ranging from 1,000 to 1,500 Å^2^), the fraction of initial contacts frequently drops under 80%, and as low as 50% for complex 1PVH (Interleukine six receptor/leukemia inhibitory factor). Interestingly, some complexes display distinct conservation levels during the simulation, suggesting the existence of distinct interface substates, like the barnase/barstar complex 1BRS, for which the fraction of initial contacts fluctuates around 90% during the first 125 ns of simulation and then around 75% for the rest of the simulation. The existence of distinct substates at the interface is also suggested by the change in the absolute number of interface contacts along MD trajectories (Supplementary Figures S8A,C).

In addition, we characterized the type of interface interaction, by categorizing each contact based on the nature of the amino acids in interactions (i.e., polar or non-polar), see Supplementary Figure S8B. For complex 1BRS, the decrease of initial contacts is accompanied by an increase in the fraction of contacts between polar residues, and a decrease for mixed polar/apolar contacts. The same tendency is observed for complex 1PVH, and for complex 1AK4, which has a noticeably higher fraction of apolar contacts compared to former complexes. We could not observe a general trend. Complex 1AY7, for example, shows an opposite behavior with a decrease in the proportion of polar contacts and an increase for mixed contacts. This suggests that the interface rearrangements observed can modify the nature of the interfaces in a case-dependent fashion.

#### 3.3.2 Protein-protein interfaces visit several substates

**FIGURE 4:**
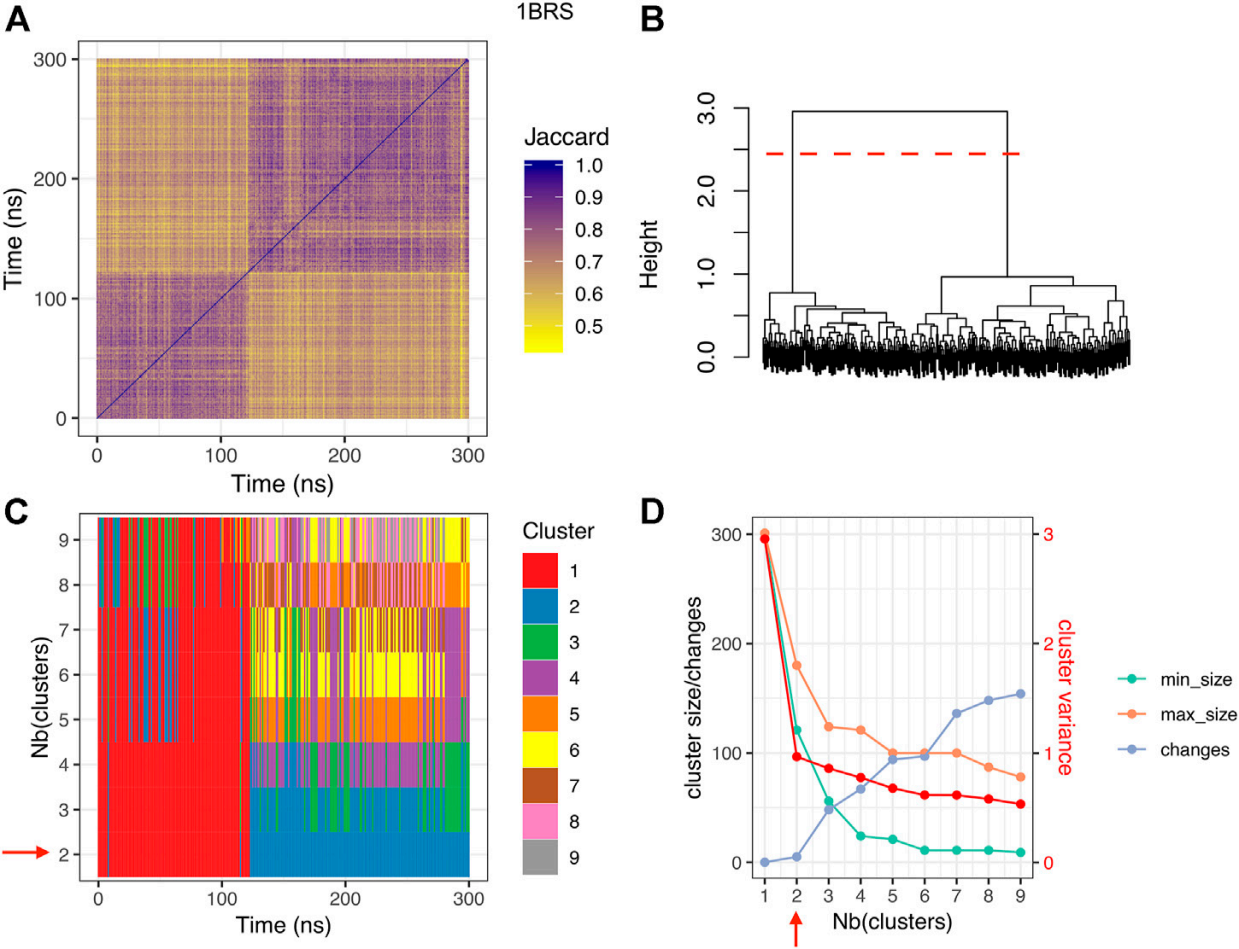
Clustering results for the barnase/barstar complex 1BRS. **(A)** Jaccard similarity matrix; for each pair of snapshots, a yellow pixel indicates low interface similarity, and a purple pixel indicates high interface similarity. **(B)** clustering dendrogram. **(C)** cluster membership along simulation time, for different numbers of clusters. **(D)** cluster size, number of changes, and intra-cluster variance (red), for different numbers of clusters; min_size: size of the smallest cluster, max_size: size of the largest cluster, changes: number of cluster changes during the simulation. The red dashed line in panel **(B)** and the red arrows in panels **(C,D)** indicate the optimal number of clusters.

To investigate the existence of substates at the interface, we computed the similarity between interfaces along the trajectory *via* the Jaccard index *J* based on interface contacts and performed hierarchical clustering, as explained in the Material and Methods section. This analysis revealed the existence of distinct substates at the interfaces. Clustering results are shown in Figure 4 for the barnase/barstar complex 1BRS, and Supplementary Figures S9–S15 for other complexes. In the case of the barnase/barstar complex 1BRS, the Jaccard similarity matrix displays two purple squares along the diagonal with yellow off-diagonal rectangles, indicating two portions of the trajectory (0–125 ns and 125–300 ns) with high internal interface similarity and well distinct from each other (Figure 4A). This is confirmed by the dendrogram, with two long branches (Figure 4B). The two main clusters correspond to long-lived and well-populated substates (Figure 4C), and adding more clusters would introduce short-lived, low-populated substates, intercalated with the previous ones (Figures 4C,D). Remarkably, we found several clusters in six out of the eight complexes studied. Confirming our observation about the interface conservation in the previous paragraph, only two complexes 2OOB (ubiquitin/ubiquitin ligase complex) and 1GCQ (Vav/GRB2 SH3 domains complex), could not yield interface clusters. By contrast, for all the other complexes, several clusters could be seen, corresponding to distinct substates due to variation of interface contacts. The number of clusters (see Supplementary Table S1) is not related with the interface size: complex 1AK4 (cyclophilin/HIV capsid) with an interface size of 1,066 Å^2^ has 4 clusters, whereas complex 1BRS (barnase/barstar) with a large interface (1,555 Å^2^) has only two clusters, even when the simulation is extended up to 500 ns (data not shown). In the next section, we further explore the structural differences between substates.

#### 3.3.3 Substates involve variability of contacts in interface cores

**FIGURE 5:**
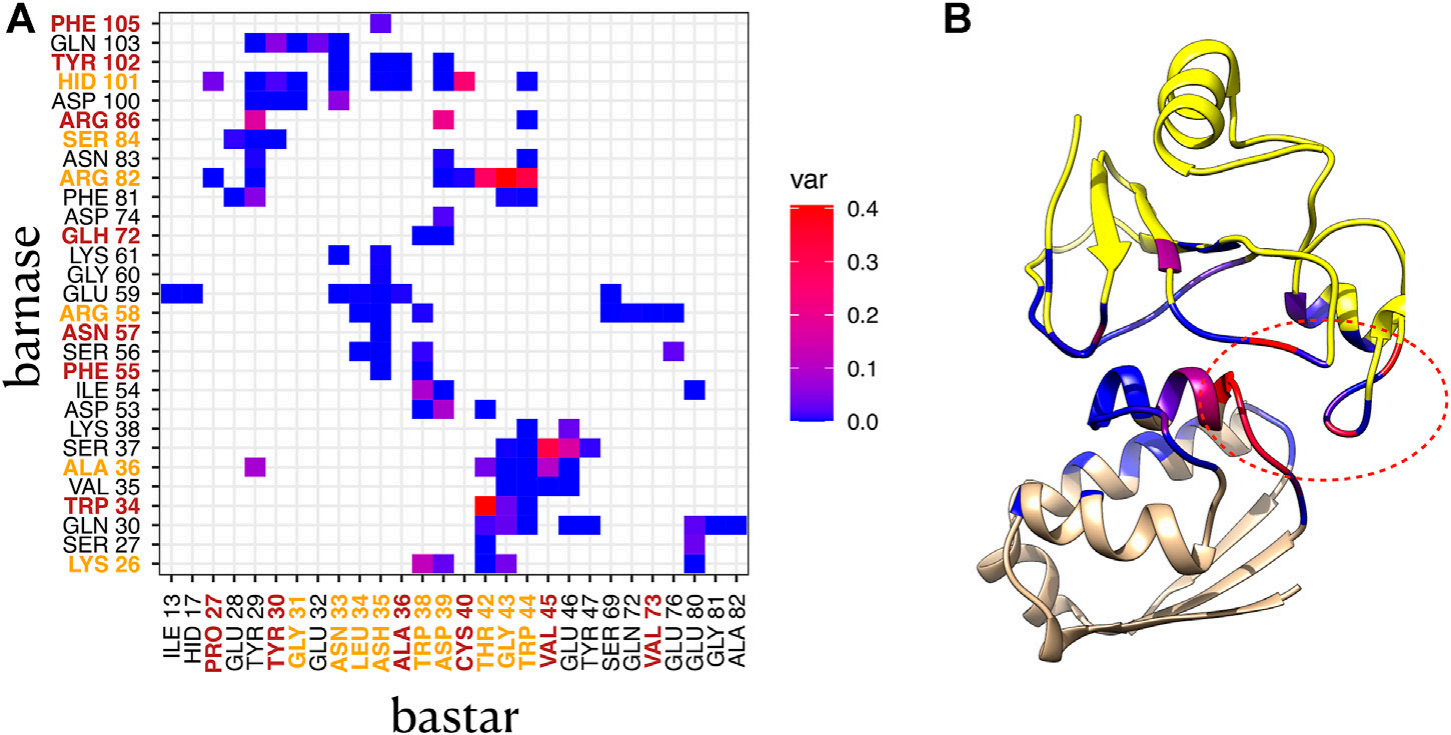
Contact variance in complex 1BRS. **(A)** variance of each contact frequency across the two interface clusters. Residues labeled in orange are core residues, and residues labeled in brown are support residues in the crystallographic structure. Protein1: barnase, protein 2: barstar. **(B)** 3D structure of 1BRS, with barnase in yellow, and barstar in beige, and interface residues colored by contact variance (maximum variance per residue). The red ellipse indicates the variable region of the interface.

To highlight the contacts that are responsible for the differences between clusters, we computed the variance of the relative frequencies of interface contacts. Figure 5A displays the contact variances for the complex 1BRS in a matrix representation. A blue square in the matrix indicates a low variance, i.e., a contact with stable relative frequency among the different clusters. On the contrary, a red square indicates a high variance, i.e., a contact whose relative frequency is different across clusters. In the case of the barnase/barstar complex 1BRS, a restricted number of contacts are actually responsible for the difference between clusters (in agreement with contact conservation in Supplementary Figure S8), while a majority of contacts have stable frequencies across clusters, see Figure 5A. These contacts involve a small set of residues: Arg82, Ser37, and Trp34 of barnase, and Thr42, Gly43, and Trp44 of barstar. Those residues are located in a restricted region of the interface, as indicated by the ellipse in Figure 5B. Also, it is worth noting that some of the residues associated with high contact variability (Arg82, Thr42, Gly43, and Trp44) are classified as interface core residues in the experimental structure, see Figure 5A and Supplementary Table S2.

The same analysis was also carried out for the other five complexes with more than one cluster. In the majority of them, a few contacts, involving two to six residues per protein, are found responsible for the difference between clusters, see Supplementary Figures S16, S17 and Supplementary Table S3. A notable exception is the complex 1AK4, where the contact variability affects all the interface contacts. It is important to point out that all the six complexes under study display variable contacts involving core residues (see Supplementary Figure S16; Supplementary Table S2). All these results highlight the need to further characterize the structural difference between substates, using representative structures of each cluster.

#### 3.3.4 Characterization of interface substates

##### 3.3.4.1 Interface properties

**FIGURE 6:**
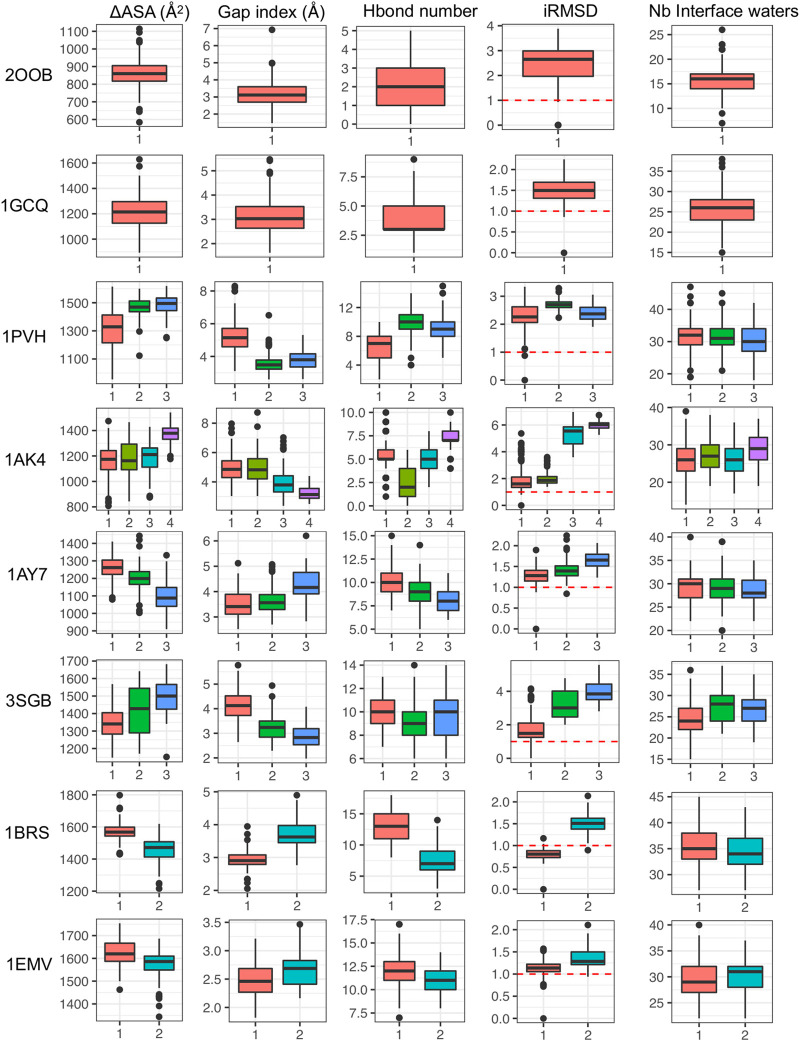
Interface properties in each cluster based on protein-protein contacts represented *via* box plots: interface size (∆ASA), interface shape complementarity (Gap index) and number of interface H bonds, iRMSD and number of interfacial water molecules.

In this section, we characterize the different interface substates visited along MD simulations in the different systems. For this, we considered interface properties and structure representatives in each cluster. Average properties computed for each interface cluster are reported in Supplementary Table S3 and distributions are shown in Figure 6 and Supplementary Figures S18, S19 (complexes with only one cluster are also integrated for the sake of completeness). For a given protein-protein complex, the different clusters have distinct average properties, meaning that the substates differ in terms of interface size (∆ASA), interface shape complementarity (Gap index) and number of interface H bonds, as shown in Figure 6. Interestingly, all the systems studied visit substates with iRMSD greater than 1 Å, which is the cutoff used to define high quality models in the CAPRI standards (Duan et al., 2020).

##### 3.3.4.2 Structure of the interface

To analyze the structural differences between interfaces clusters, we extracted representative structures as explained in the Material and Methods section. For each molecular system, we superimposed the representative structures of the different clusters to highlight global changes. We also visualized the interfaces and made the distinction between 1) interface residues that preserve the same contacts in the different clusters, 2) interface residues that make different contacts and 3) interface residues that are specific to some clusters. We also included interface waters in the visualization.

**FIGURE 7:**
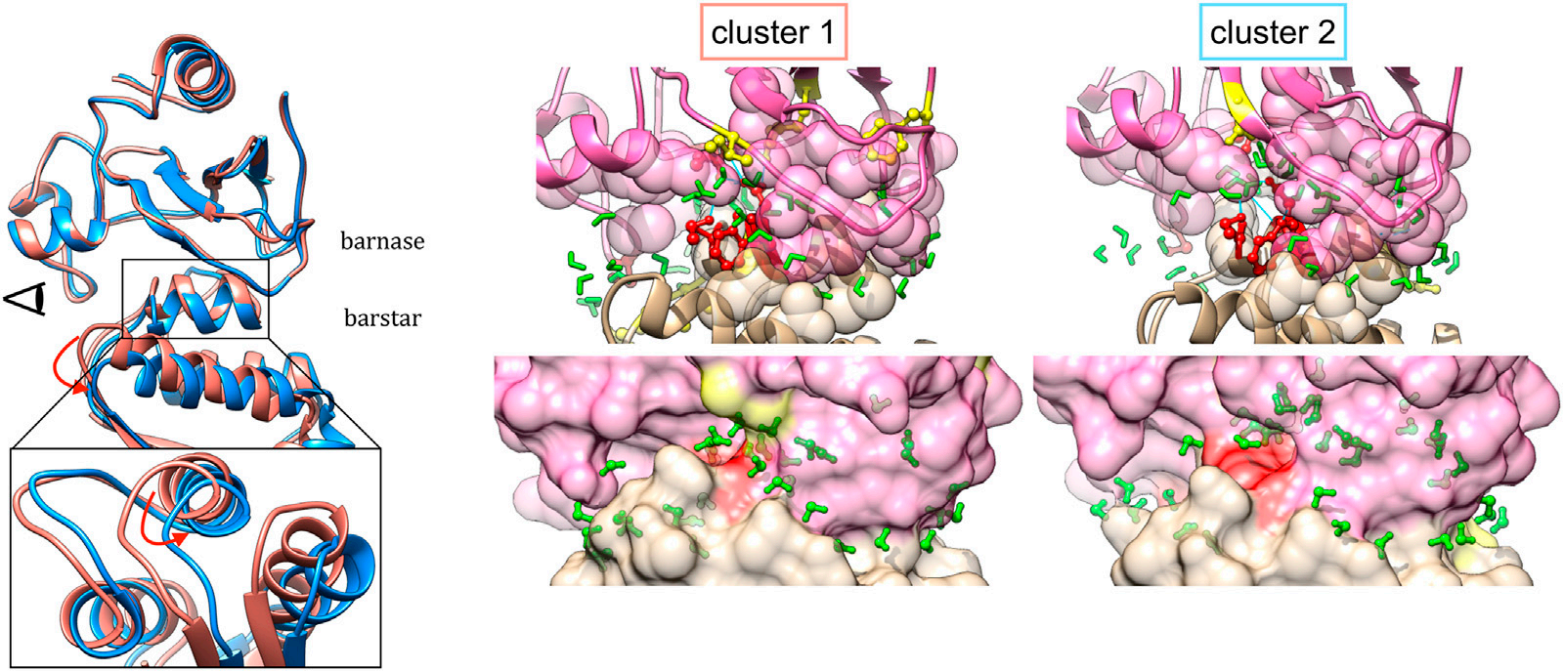
Representative structures of interface clusters for the barnase/barstar complex 1BRS. Left part: global superimposition of structure representatives, with cluster one in pink and cluster two in blue and red arrows pointing at main structural variations. The eye symbol indicates the point-of-view for the close-up view at the bottom, where barnase is omitted for clarity. Middle and right part: structure of interfaces in each cluster, with barnase in pink and barnase in beige, and water molecules in green. Interface residues are represented as follows: residues involved in stable contacts as spheres, residues involved in variable contacts as red balls and sticks, residues involved in contacts that are specific to one cluster in yellow balls and sticks.

The representative structures in the case of the barnase/barstar complex 1BRS is shown in Figure 7. The comparison of representative structures reveals a slight rotation of barnase with respect to barstar, which opens the left side of the interface, with some contacts that are rearranged (red residues) or lost (yellow residues). This results in a smaller interface (∆ASA equal to 1,573 Å^2^ in cluster one versus 1,459 Å^2^ in cluster 2). As binding affinity is linked to the interface size, the two clusters presumably have different binding affinities. The existence of substates with lower affinity could assist in the reversibility of the protein-protein interaction.

Representative structures for substates in other complexes are shown in Supplementary Figures S20, S21. The RNase/barstar complex 1AY7, (Supplementary Figure S20A), shows a slight rotation of RNase with respect to barstar, similar to 1BRS, with also the rearrangement of a loop at the interface. The IL receptor/inhibitory factor complex 1PVH (Supplementary Figure S20B), also reveals a rotation of one protein with respect to the other. The interface is rearranged in a way that maintains the central contacts and results in a larger and more compact interface.

As shown earlier, the cyclophilin/HIV capsid complex 1AK4, undergoes a significant reorientation of the two proteins during the simulation time, which can be seen on the structure representatives (see Supplementary Figure S20C). The streptogrisin/inhibitor complex 3SGB (Supplementary Figure S21A) also displays a substantial change of orientation between the two proteins, although less dramatic. However, in this case, a majority of contacts are stable in the middle of the interface. In the colicin endonuclease/inhibitor complex 1EMV (Supplementary Figure S21B), the substates differ by a small loop movement the inhibitor, leading to the rearrangement of a few contacts, including those formed by an aspartate residue of the endonuclease in the core of the interface.

#### 3.3.5 Comparison of interface contacts dynamics with mutation data

We compared our results on interface contacts dynamics with available data for binding affinity changes upon mutations from the SKEMPI v2.0 database (Jankauskaitė et al., 2019). Four of the studied complexes were present in SKEMPI, for a total of 82 experimental measures of binding affinity change. The comparison of binding affinity changes with the involvement of residues in interface substates is shown in Supplementary Figure S22. We observed that interface hotspots are exclusively residues with high recurrence index (see Eq. 5), i.e., residues that are part of the interface in all clusters. Concerning variance of contact frequencies, hotspots span a wide range of maximum variance (see Eq. 4), meaning that they can be involved in contacts that have different frequencies between clusters. This suggests that hotspot residues are not necessarily involved in invariant contacts in protein-protein interfaces but could form different sets of contacts in different substates.

### 3.4 Water analysis: Non-polar solvation energy and interfaces

To better characterize the interfaces and the complexes, we also analyzed the role of water from an energetic and a structural point of view at the interface and in the formation of the complex.

#### 3.4.1 Water and non-polar solvation energy

**FIGURE 8:**
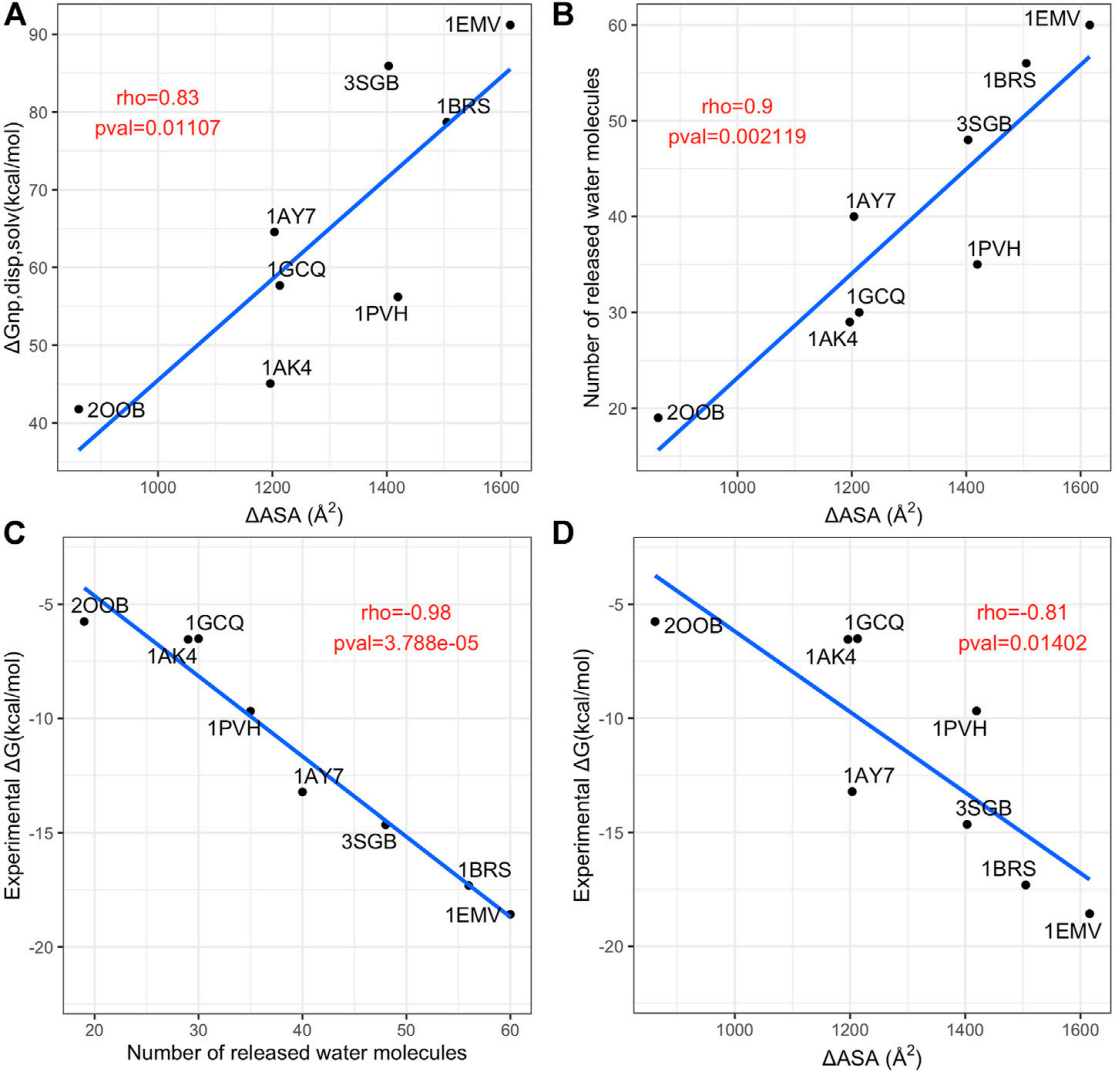
Analysis of contributions to the non-polar solvation energy. **(A)** Scatterplot of the interface size (*x* axis) versus dispersive solvatation energy, ∆G_np,disp, solv_ (*y* axis). **(B)** Scatterplot of the interface size (*x* axis) versus number of released water molecules. **(C)** scatterplot of the number of released water molecules (*x* axis) versus the experimental binding affinity (*y* axis). **(D)** scatterplot of interface size (*x* axis) versus the experimental binding affinity (*y* axis). In each panel, the blue line is the linear regression fit and the Pearson rho coefficient and associated *p*-values are annotated in red.

We computed the dispersive solvation energy, ∆*G*_*np*,disp, solv_, and the number of water released at the first and second shell, as explained in the Material and Methods section. Then, we analyzed the relationship between the number of water molecules released at the first shell and ∆ASA and the binding affinity. Figure 8 summarizes these data. Although in usual MM-PBSA approaches (Hou et al., 2011; Wang et al., 2018), the non-polar solvation energy component term is usually calculated *via* linear equation with respect to ASA of the protein and protein complexes, it turned out that there is no simple relationship between ∆ASA and the dispersive solvation energy, ∆*G*_*np,*disp, solv_, see Figure 8A. This finding supports the fact that the standard ASA model does not work properly as pointed out by other groups who proposed to further decomposed the nonpolar component into attractive (dispersive) and repulsive (cavity) components using the Weeks-Chandler-Anderson (WCA) separation scheme (Weeks et al., 1971; Chandler et al., 1983; Levy et al., 2003; Zacharias, 2003; Shivakumar et al., 2009):

$$∆G_{np,solv}=∆G_{np,disp,solv}+{∆G}_{np,cavity,solv}$$ (6)

where ${∆G}_{np,cavity,solv}$ is the cavity hydration free energy that takes into account the reorientation of the water molecules. However, the latter takes only into account the change of ASA and ignores the entropy of transfer of water molecules from interfacial surfaces to the bulk. Here, we show that there is not a simple relationship between ∆ASA and the number of water molecules released in the first shells, see Figure 8B, highlighting the limit of the ${∆G}_{np,cavity,solv}$ term. Moreover, the number of released water molecules displays a very high correlation with the binding affinity, see Figure 8C, with a correlation coefficient equal to −0.98. This correlation is significantly higher than the correlation between interface size and binding affinity (see Figure 8D, rho = −0.81) according to the Dunn and Clark test (pval = 0.0002).

#### 3.4.2 Structural interfacial waters

##### 3.4.2.1 Evolution of interfacial waters

First, for each representative structure we determined the number of interfacial waters and we observed that there are no significant changes along the clusters, unlike the other interface descriptors (see Figure 6). In fact, the number of water molecules that are close to the interface remain generally quite stable, despite the possible variations of interface size. The stability of the total amount of interface water molecules hides the structural rearrangement of water molecules at the interface since water dynamics is much faster. On the contrary, if we analyze the location of these water molecules at the interface, they differ between clusters (see Figure 7 for the barnase/barstar complex 1BRS). In the next paragraph, we further quantify these variations. These results allow us to speculate that the dynamics of the interfacial waters for the different clusters may have different dynamics.

##### 3.4.2.2 Contacts between interface residues and interface water molecules

**FIGURE 9:**
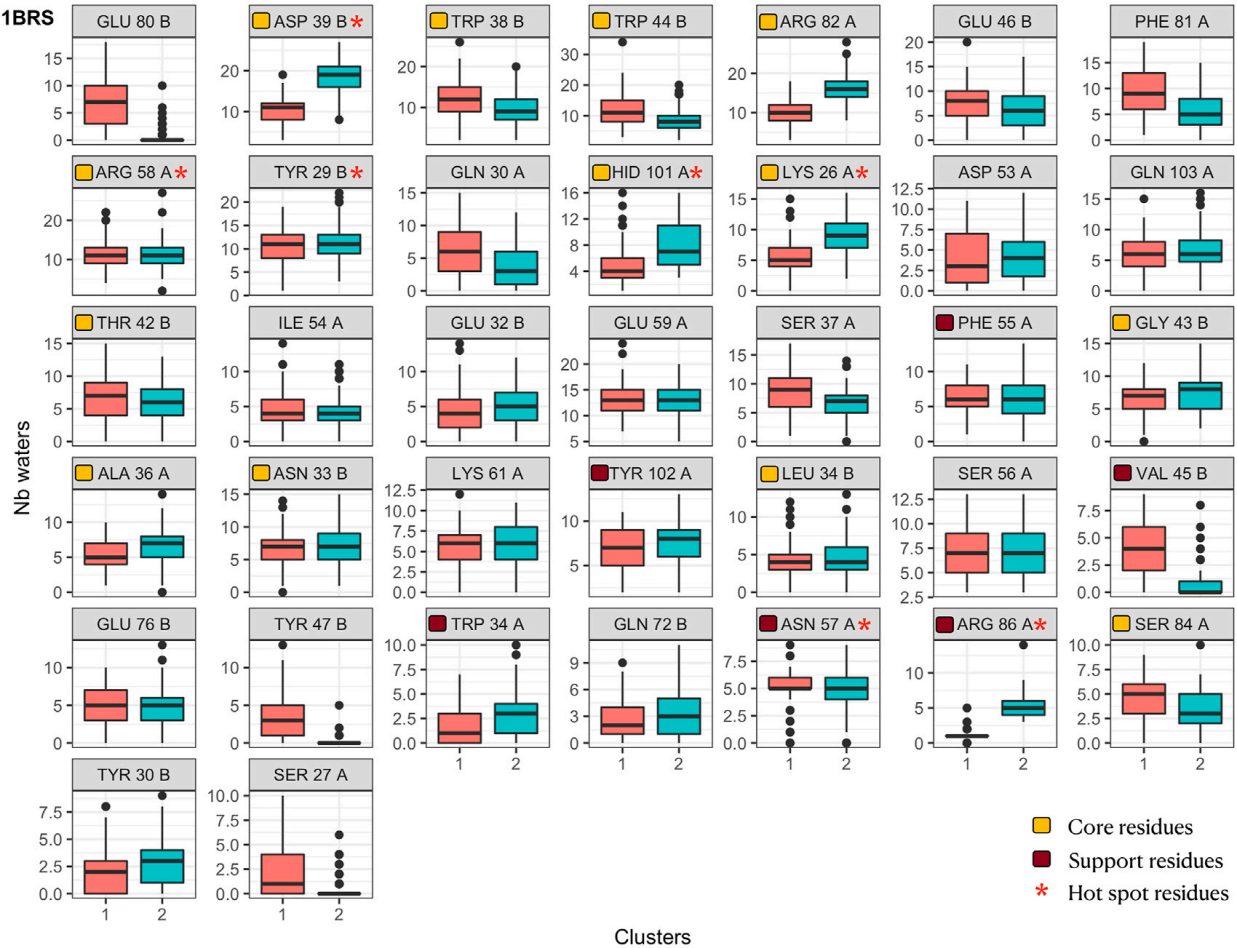
Number of interface water molecules in contact with each interface residue in the barnase/barstar complex 1BRS. Distributions are shown as boxplot in each interface cluster. Orange squares indicate core residues and brown squares indicate support residues (in X-ray structures), as in Figure 6. Red stars indicate hotspot residues.

We analyzed the contacts between interface residues and interface water molecules and confronted those results with the interface clustering. Figure 9 displays, for the barnase/barstar complex 1BRS, the number of interface water molecules in contact with each interface residue during the simulation, colored by interface cluster membership. For clarity, only the residues with variable amounts of water contacts (standard deviation greater than 2) are plotted. Similarly to what we observed for interface contacts (see Figure 5), residues with a variable number of interface water contacts are not restricted to the periphery of interfaces but also include core and support residues. Interestingly, variable residues also include hotspot residues. This figure clearly highlights the residues with different solvation states between clusters: for example, Glu80 in barstar is more solvated in cluster one and Asp39 is more solvated in cluster 2. Other residues have a variable number of contacts with interface water molecules without a clear correlation with interface clusters, like Arg58 of barnase. The analyses for other complexes are shown in Supplementary Figures S23–S27. This analysis offers us yet another way to picture interfaces, as dynamical objects that can rearrange their contacts both between residues from interacting chains and water molecules.

##### 3.4.2.3 Contacts mediated by water molecules at the interface

To better characterize the contacts with water and their impact on the interactions, we also analyzed the contacts that are mediated by water molecules at the interface, i.e., triplets constituted by a residue from one chain in contact with a water molecule which is in contact with a residue from the other chain. A water-mediated contact between two residues is then defined by the existence of such triplet in the snapshots. The connecting water molecule can change between snapshots since its dynamics is much faster than the time between each snapshot. Since the study of the dynamics of water is beyond this work, we only assess the stability of the residue pairs. We computed the relative frequency of such water-mediated contacts in each complex, also taking into account the location of the residues in the different regions of the interfaces (core or periphery). These results are shown in Supplementary Figure S28. First, for all complexes several water-mediated contacts are observed at their interface that can involve residues that are less exposed in the experimental structures, i.e., classified as core or support residues. We also observe that some of these contacts are fairly stable during the simulation time, appearing 75% of the time or more, underlying the structural role of interface waters. Lastly, many contacts involve charged residues, sometimes with charges of the same sign, showing the screening effect of water molecules that allow the presence of charged residues at the protein interface and stabilize the interaction of charged residues of the same charge. In order to understand how the loss of initial contacts could be linked to water molecules, we also computed the number of water-mediated contacts along simulation time (see Supplementary Figure S8C). We observe that decrease of initial contacts is only in part due to the loss of contacts mediated by water molecules. Concerning the total number of water-mediated contacts along simulations, it follows the same evolution as the number of direct residue-residue contacts (see Supplementary Figure S8A). The exception is 1AK4: on the one hand a decrease of the number of contacts is observed and on the other hand an increasing number of contacts mediated by water is obtained. This inverse trend is due to the opening of the interface allowing more water molecules to intercalate between the two proteins.

**FIGURE 10:**
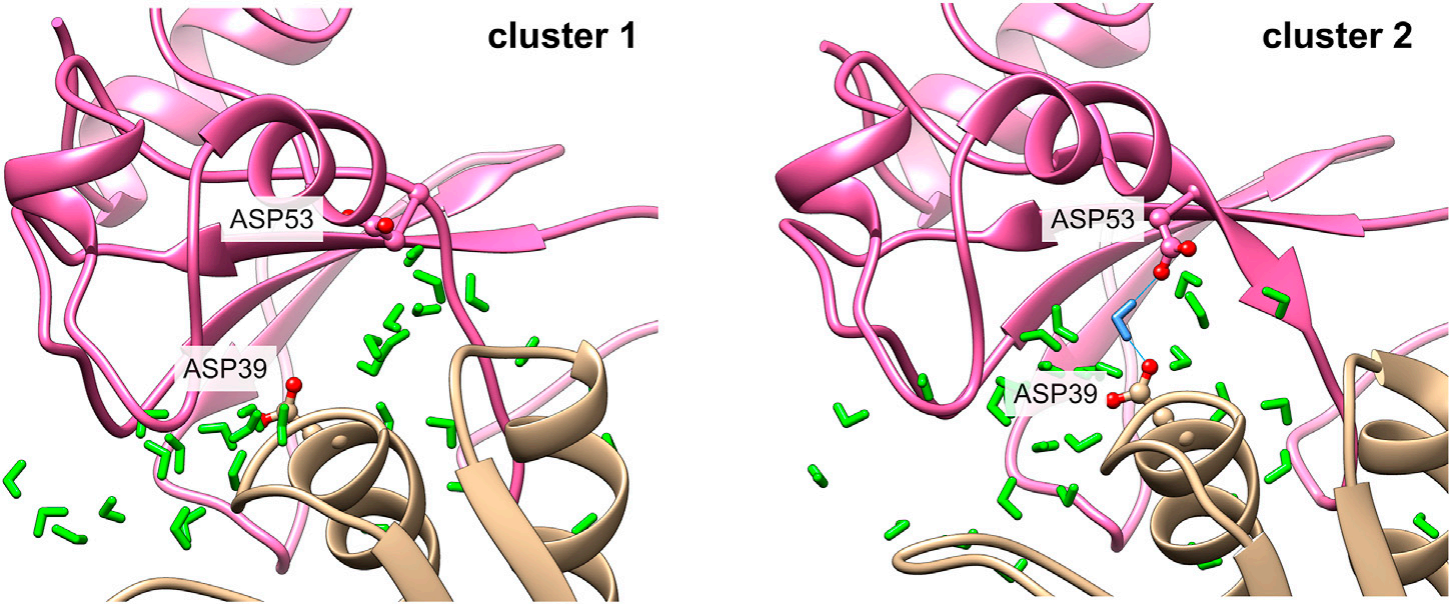
Water-mediated contact in the barnase/barstar complex 1BRS. The contact between Asp 53 of barnase and Asp 39 in barstar is mediated by a water molecule in cluster 2 (right side), with the mediating water molecule in blue. This contact is not observed in cluster 1 (left side).

Figure 10 shows an example of a water-mediated contact at the interface of the barnase/barstar complex. A water-mediated interaction between two aspartate residues (Asp 53 of barnase and Asp 39 of barstar) is observed during 64% of the simulation. As can be seen in Figure 9, the interaction of Asp 39 of barstar with interface water changes depending on the interface clusters. This water-mediated interaction is indeed specific to the second cluster observed after the first 125 ns of simulation. Of note, this second cluster has a smaller interface and a lower number of interface contacts compared to the initial cluster, but the number of interfacial waters is similar in both clusters (see Figure 6). The number of water-mediated contacts follows the same trend as the number of interface contacts, i.e., a lower number of water-mediated contacts in the second cluster. This indicates that the loss of contacts is not compensated by an increase in the number of water-mediated contacts. Rather, a rearrangement of interfacial waters allows to maintain a stable number of interfacial water molecules around interfaces of different sizes.

## 4 Discussion

Protein-protein interfaces can no longer be considered as static objects, but their dynamical nature has to be taken into account to give a more complete picture of protein-protein complexes. The present work aimed at describing what is the dynamical fate of protein-protein interfaces. We observed that at the scale of hundreds of ns, most protein-protein complexes, beyond a certain interface size, visit distinct interface substates. Interface substates are characterized by alternate interface contacts, direct or mediated by water molecules, with interface waters accompanying these rearrangements and modulating the hydration states of residues. It is important to note that these changes are not confined to the periphery of the interfaces, but also affects residues of the interface core. Either geometric or physio-chemical interface properties between substates are significantly affected in a case-dependent fashion, which could result in different complex stability and help the interaction reversibility.

Our work can be put in relation to other studies of protein-protein complexes by MD. Simulations of the association process of protein-protein complexes at the sub-millisecond scale by Pan et al. (2019) allowed the observation of transition states preceding the association, with no more than 20% of the native contacts formed, and largely hydrated interfaces. Native complexes did not dissociate once formed. Here, we observe interface rearrangement around the native state into stable clusters, starting from crystallographic structures, on a timescale of hundreds of ns. The interface substates are thus distinct from the transition states observed by Pan et al. (2019).

In a recent work, Jandova et al. (2021) studied the stability in MD of native and non-native docking models for 25 complexes, to distinguish them. They observed higher stability of interface properties for native models, and also noticed significant changes in simulations starting from crystallographic structures in their simulations of 100 ns. In another recent work, Prévost and Sacquin-Mora performed MD simulations on docking models of various quality for three complexes submitted at the CAPRI competition and observed a category change (in terms of model quality) for more than half of the models (Prévost and Sacquin-Mora, 2021). Crystallographic structures stayed in the medium quality range in their 100 ns simulations and preserved more than 50% of their native contacts. In the present study, the transition to a new interface cluster generally happened after the first 100 ns. So, our observations of substates probably apply to the time range in between these recent studies and the very long simulations of Pan et al. (2019) Nevertheless, they all go in the same direction, in the realization that protein-protein interfaces are dynamical too. Let us recall here that we simulated only complexes that behave as rigid bodies, so rigid bodies do not equal static objects. As observed in recent works (Jandova et al., 2021; Prévost and Sacquin-Mora, 2021), the protein-protein complexes visited conformations outside the high quality range of CAPRI, suggesting that docking model evaluation should take this variability into account.

Our work also highlights the crucial role of water in the binding and in the strength of the interactions, in terms of the number of released water molecules and the number of contacts mediated by water molecules at the interface. In this context, we observed that the number of released water molecules at the first shell is strongly correlated to the experimental binding affinity. Hence, first, our results suggest that for MM-PBSA approaches it is recommended to take into account the simulations of the unbound and bound states (3 simulations). Secondly, a new way to describe the non-polar solvation energy seems crucial in MM-PBSA approaches and in protein-protein docking based on physical scores. Finally, the amount of water-mediated contacts observed in the simulations of the complexes suggests that in protein-protein docking the presence of water molecules at the interface may open a new route to predict them.

## Data Availability

The original contributions presented in the study are included in the article/Supplementary Materials and MD simulations without water molecules and ions for the unbound proteins and with water molecules for the complexes generated for this study can be found in the repository Zenodo (doi: 10.5281/zenodo.6638504).
