# Supplementary material for: A dynamical view of protein-protein complexes: Studies by molecular dynamics simulations
Source: Front Mol Biosci. 2022 Oct 6;9:970109. doi: 10.3389/fmolb.2022.970109 (PMC9583002; doi:10.3389/fmolb.2022.970109)
Supplement: Supplementary file 1 [file DataSheet1.PDF]

frontiers

Supplementary Material

A dynamical view of protein-protein complexes : studies by molecular dynamics simulations

Juliette Martin[1*], Elisa Frezza[2*]

[1]Univ Lyon, CNRS, UMR 5086 MMSB, 7 passage du Vercors F-69367, Lyon, France

[2]CiTCoM, CNRS, Université Paris Cité, Paris, France.

*** Correspondence:**
Corresponding Authors
juliette.martin@ibcp.fr

elisa.frezza@u-paris.fr

Supplementary Tables

Supplementary Table 1. Data set description. Abbreviations used: ITC: Isothermal Titration Calorimetry, SPR: Surface Plasmon Resonance, FIA: Fluorescence Inhibitory Assay, SIA: Spectrophotometric Inhibition Assay, SF: Stopped-flow Fluorimetry. CT: C-terminal missing. NT: N-terminal missing. The number of interface substates (last column) is deduced from the interface clustering analysis of the present study.

Complex	Protein 1	Protein 2	△G (kcal/mol)	Method	△ASA in the X-ray structure (Å^2)	Number of interface contacts in the X-ray structure	Number of interfaces substates in the simulation
2OOB_A:B	2OOA_A, Ubiquitin ligase	1YJ1_A, Ubiquitin	-5.66	ITC	808	28	1
1GCQ_B:C	1GRI_B, GRB2 C-ter SH3 domain	1GCP_B (NT), Vav N-ter SH3 domain	-6.51	SPR	1207	49	1
1AY7_A:B	1RGH_B, RNase Sa	1A19_B, Barstar	-13.23	FIA	1227	41	3
1PVH_A:B	1BQU_A (CT,NT), IL6 receptor beta chain D2-D3 domains	1EMR_A (NT), Leukemia inhibitory factor	-9.52	ITC	1403	49	3
1AK4_A:D	2CPL_A, Cyclophilin	1E6J_P(CT), HIV capsid	-6.43	ITC	1066	50	4
1BRS_A:D	1A2P_A, Barnase	1A19_B, Barstar	-17.32	FIA	1555	55	2
3SGB_E:I	2QA9_E, streptogrisin B	2OVO_A, Ovomucoidin inhibitor	-14.51	SIA	1269	55	3
1EMV_A:B	1FSJ_B, Colicin endonucleaseE9	1IMQ_A, IM9 inhibitor	-18.58	SF	1535	56	2

Supplementary Table 2. Residues associated with variable contacts in each complex. We report the residues involved in contacts with variance greater than 0.2, ordered by decreasing variance. Residues that are underlined are classified as core or support in experimental structures; residues in italic are residues with low recurrence index.

Complex	Protein 1	Protein 2
1AY7	*ASN 39*, GLN 32	ASP 35, *GLY 43*
1PVH	THR 45, ASN 72, *ASN 92*, ALA 44, VAL 68, ASP 94, HIS 46, VAL 71, TRP 43, *PHE 93*	SER 117, HIS 6 , ILE 4 , GLY 114, ARG 5, ARG 113, ASP 110, CYS 121, LEU 116
1AK4	*HIS 54*, ARG 55, ARG 148, GLN 63, ASN 149, GLY 72, THR 73, TRP 121, *LYS 151*, HIS 126, ALA 101, ALA 103, ASN 108, *GLY 150*, LEU 122	HIS 87, PRO 85, *GLU 98*, ALA 88, VAL 86, ALA 92, *LEU 83, HIS 84*, ILE 91
1BRS	TRP 34, ARG 82, SER 37, HIS 101, ARG 86	THR 42, GLY 43, *VAL 45*, TRP 44, *CYS 40*, ASP 39
3SGB	PRO 138, GLY 121, *ASP 124*	*ASN 33, CYS 38, GLY 32, LYS 34*
1EMV	SER 49, *GLY 48*, GLU 40	LYS 89, LYS 97

Supplementary Table 3. Average property values for different interface sub-states. Average values computed in each cluster are reported with standard deviation in brackets. iRMSD is computed with the initial structure as a reference.

Complex	cluster	Nb contacts	△ASA ($Å^2$)	Gap index (Å)	Nb Hbonds	iRMSD (Å)
1AY7	1	40 (3)	1259 (62)	3.5 (0.5)	9.9 (1.4)	1.3 (0.2)
	2	38 (3)	1197 (69)	3.6 (0.5)	8.9 (1.5)	1.4 (0.2)
	3	33 (4)	1092 (91)	4.3 (0.6)	8.0 (1.2)	1.6 (0.2)
	X-ray/t=0	41/44	1237/1280	2.8/2.6	15/10	
1PVH	1	41 (5)	1315 (137)	5.2 (0.9)	6.3 (2.0)	2.3 (0.5)
	2	46 (3)	1470 (58)	3.6 (0.5)	10.0 (1.7)	2.7 (0.3)
	3	46 (4)	1481 (77)	3.8 (0.5)	9.4 (1.9)	2.4 (0.3)
	X-ray/t=0	49/45	1403/1340	4.4/3.5	8/4	
1AK4	1	44 (5)	1162 (120)	4.9 (0.9)	5.5 (1.6)	1.8 (0.8)
	2	42 (6)	1188 (137)	5.0 (1)	2.5 (1.7)	2.0 (0.5)
	3	38 (5)	1188 (117)	4.0 (1)	5.0 (1.5)	5.3 (0.8)
	4	42 (3)	1370 (76)	3.3 (0.5)	7.3 (1.4)	6.0 (0.3)
	X-ray/t=0	50/45	1066/1184	5.0/ 5.1	6/6	
1BRS	1	53 (2)	1573 (56)	2.9 (0.3)	12.8 (2.1)	0.8 (0.1)
	2	44 (3)	1459 (76)	3.7 (0.4)	7.6 (1.8)	1.5 (0.2)
	X-ray/t=0	55/52	1556/1612	2.5/2.3	15/11	
3SGB	1	58 (5)	1342 (86)	4.2 (0.6)	9.8 (1.4)	1.7 (0.7)
	2	61 (6)	1420 (136)	3.3 (0.5)	9.5 (1.8)	3.2 (0.9)
	3	64 (5)	1493 (94)	2.9 (0.5)	9.7 (1.8)	4.0 (0.7)
	X-ray/t=0	33/56	1269/1288	3.5/3.6	7/8	
1EMV	1	60 (3)	1626 (51)	2.5 (0.3)	11.9 (1.7)	1.1 (0.2)
	2	54 (4)	1568 (79)	2.7 (0.3)	10.9 (1.4)	1.4 (0.2)
	X-ray/t=0	56/64	1535/1654	2.8/2.7	12/17	

2OOB	1	29 (3)	861 (71)	3.2 (0.7)	2.0 (1.0)	2.4 (0.7)
	X-ray/t=0	28/24	808/802	3.6/2.9	3/1	
1GCQ	1	41 (5)	1213 (116)	3.1 (0.7)	3.7 (1.5)	1.5 (0.3)
	X-ray/t=0	49/37	1208/1084	3.6/3.3	10/2	

Supplementary Figures

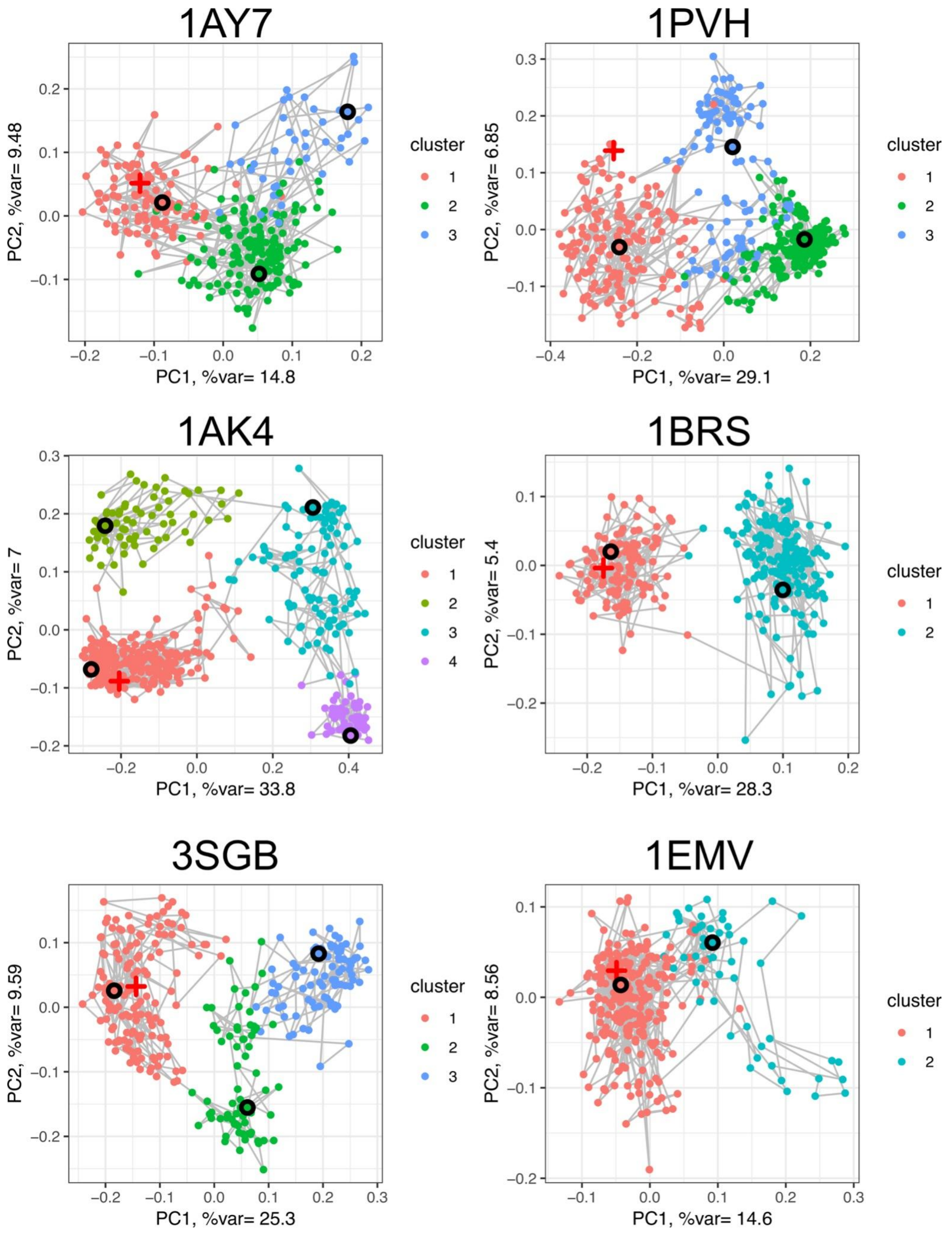

Supplementary Figure 1. Projection of snapshots and cluster centroids on the first two dimensions of the principal component analysis. Points are colored according to the cluster, connected in their order in time. Red crosses indicate the starting point of the simulations; black circles indicate cluster centroids. The percentage of variance explained by each principal component is indicated on each axis.

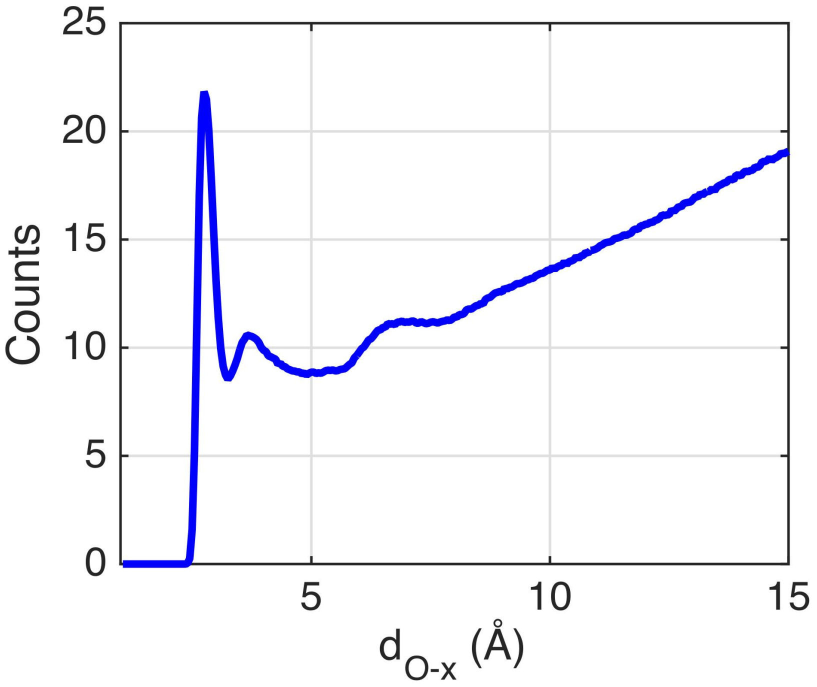

Supplementary Figure S2. Counts of number of water molecules at given minimal distance between the heavy atoms of the protein and the oxygen of the water molecules. An interval of 0.1 Å was considered for each bin.

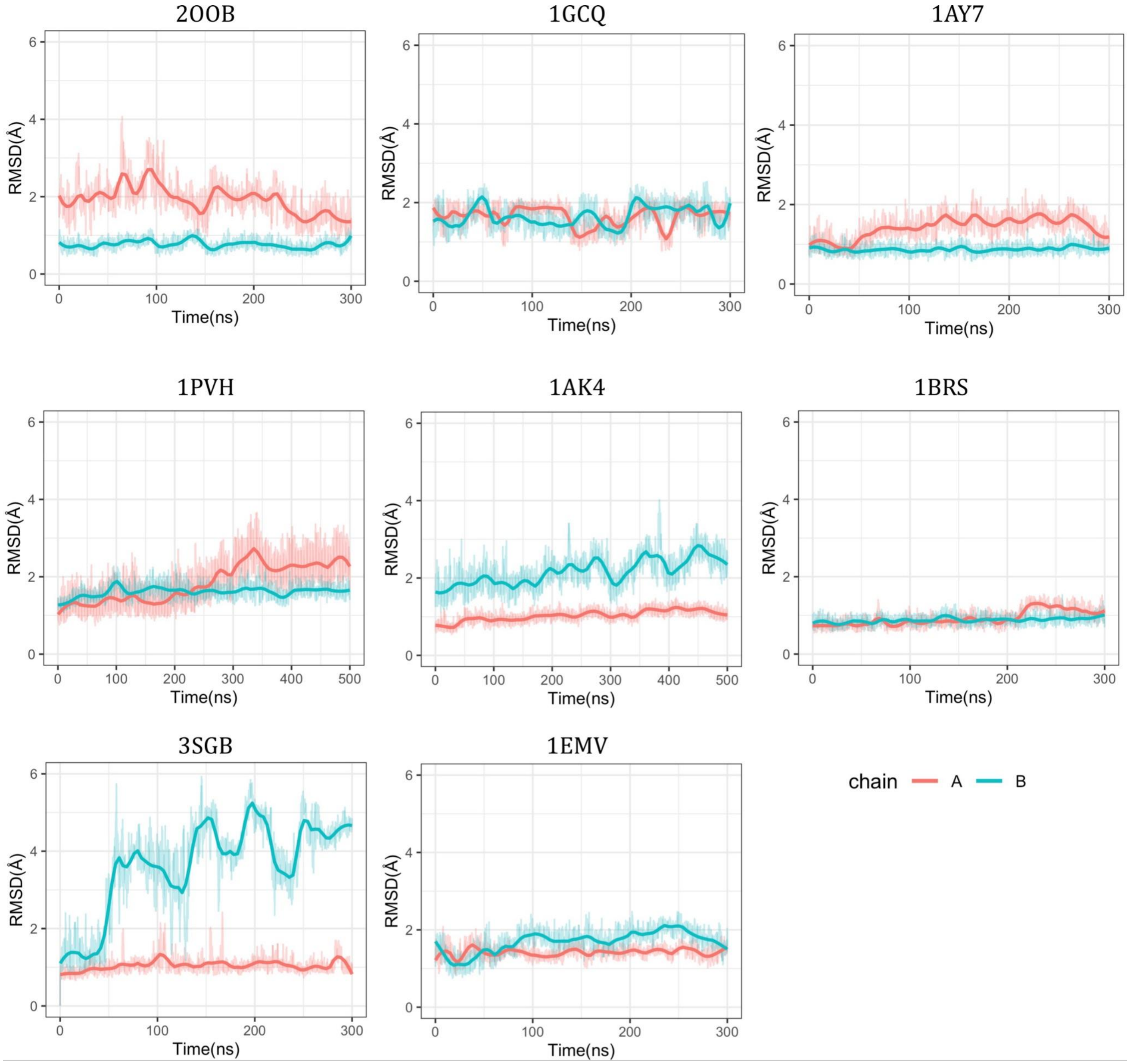

Supplementary Figure 3. RMSD time series for the simulations of the eight protein complexes. RMSDs are computed separately for each chain, with the starting structure as reference. RMSD are stable for the majority of systems, except chain B of complex 3SGB, which exhibits large RMSD fluctuations, due to a loop movement (see Supplementary Figure 4).

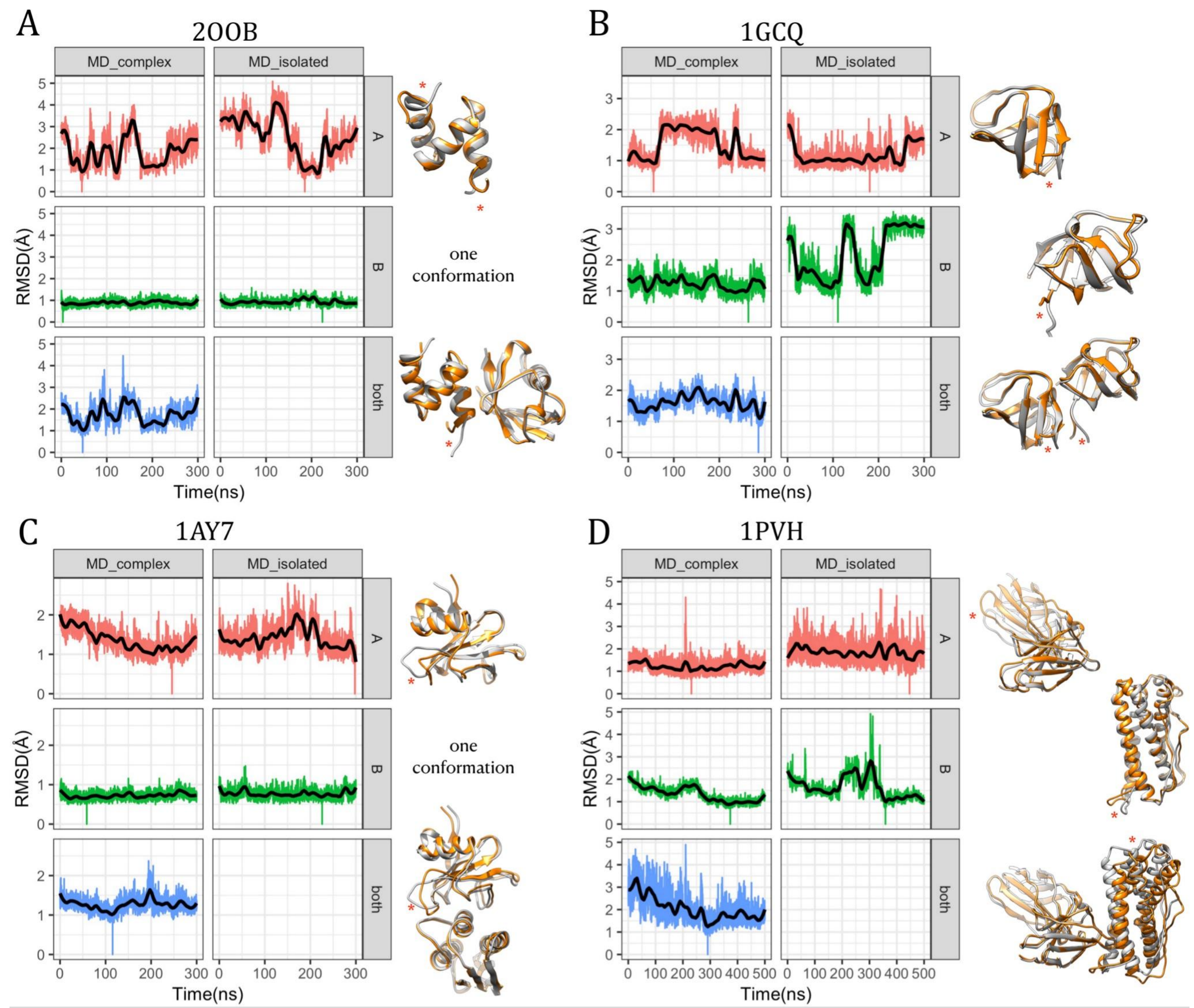

Supplementary Figure 4. RMSD cluster analysis for complexes 2OOB, 1GCQ, 1AY7 and 1PVH. RMSD time series are shown for simulations of complexes (left column), and unbound forms (right column). In each case, the RMSD is computed with the center of the main cluster as reference. Representative 3D structures are shown in cases where more than one cluster was identified, with differences highlighted by red stars.

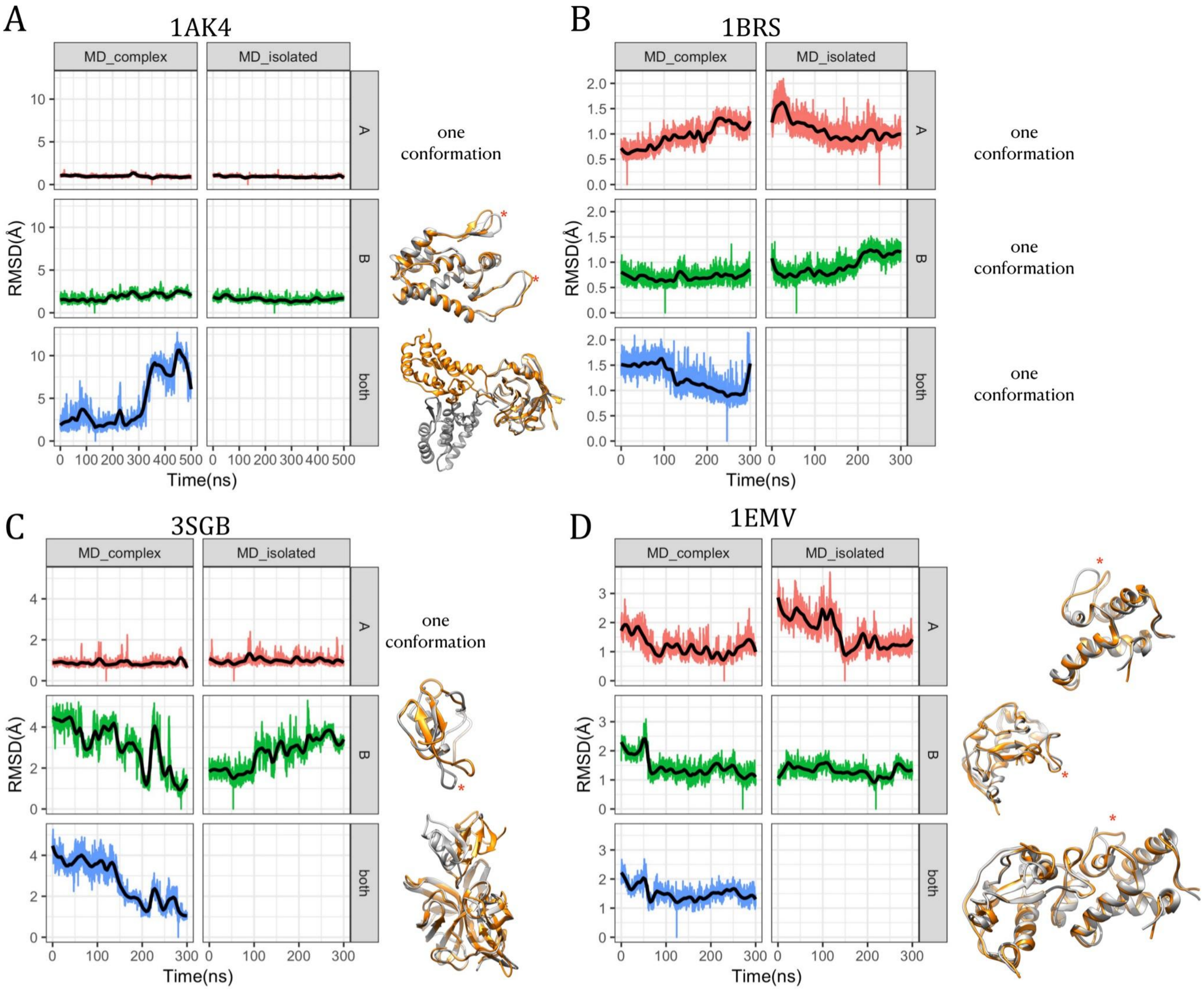

Supplementary Figure 5. RMSD cluster analysis for complexes 1AK4, 1BRS, 3SGB and 1EMV. See legend of Supplementary Figure 3 for details.

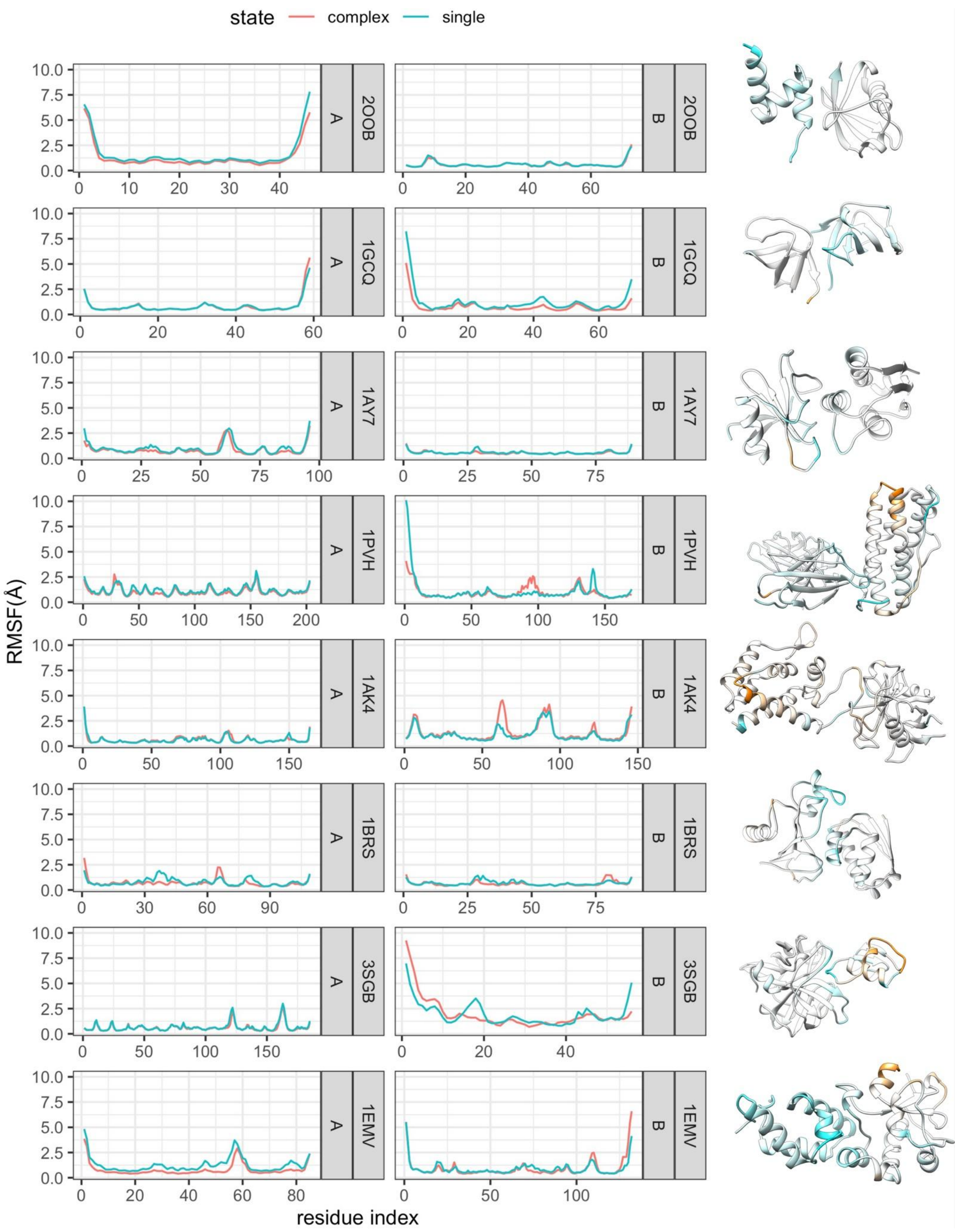

Supplementary Figure 6. Backbone RMSF profiles computed from simulations for proteins in complexes (pink) and unbound states (blue). 3D structures are colored according to the difference in RMSF, with the single proteins taken as reference, on a scale going from -1.2 (orange) to +1.2 Å (cyan).

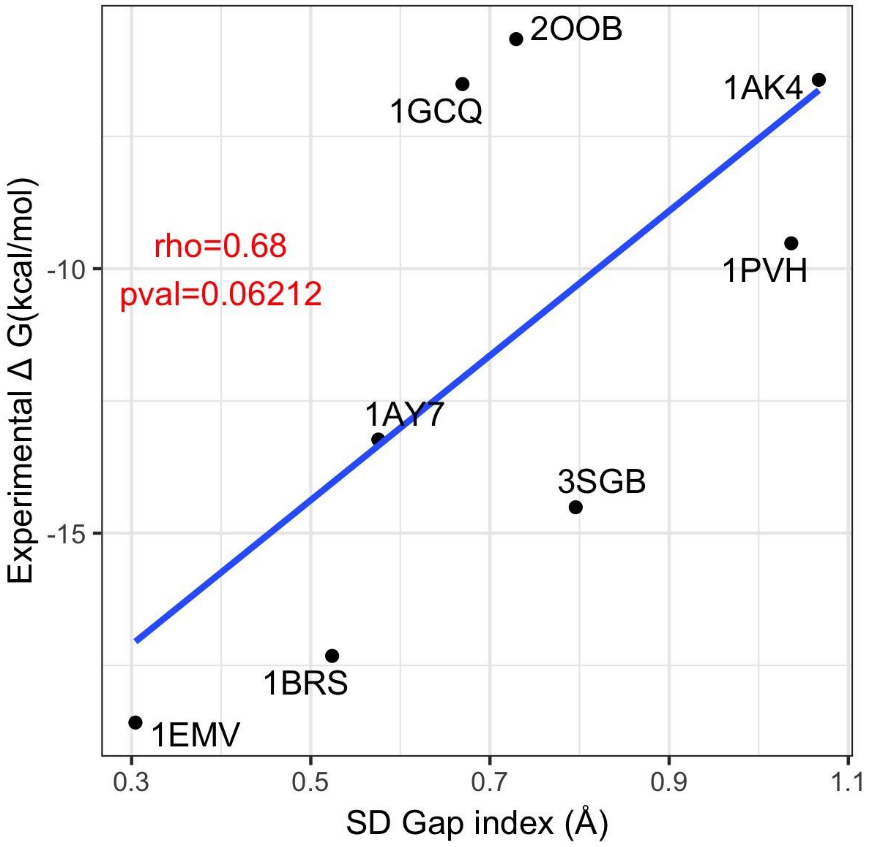

Supplementary Figure 7. Experimental binding affinity *versus* standard deviation of the gap index values sampled in the simulations.

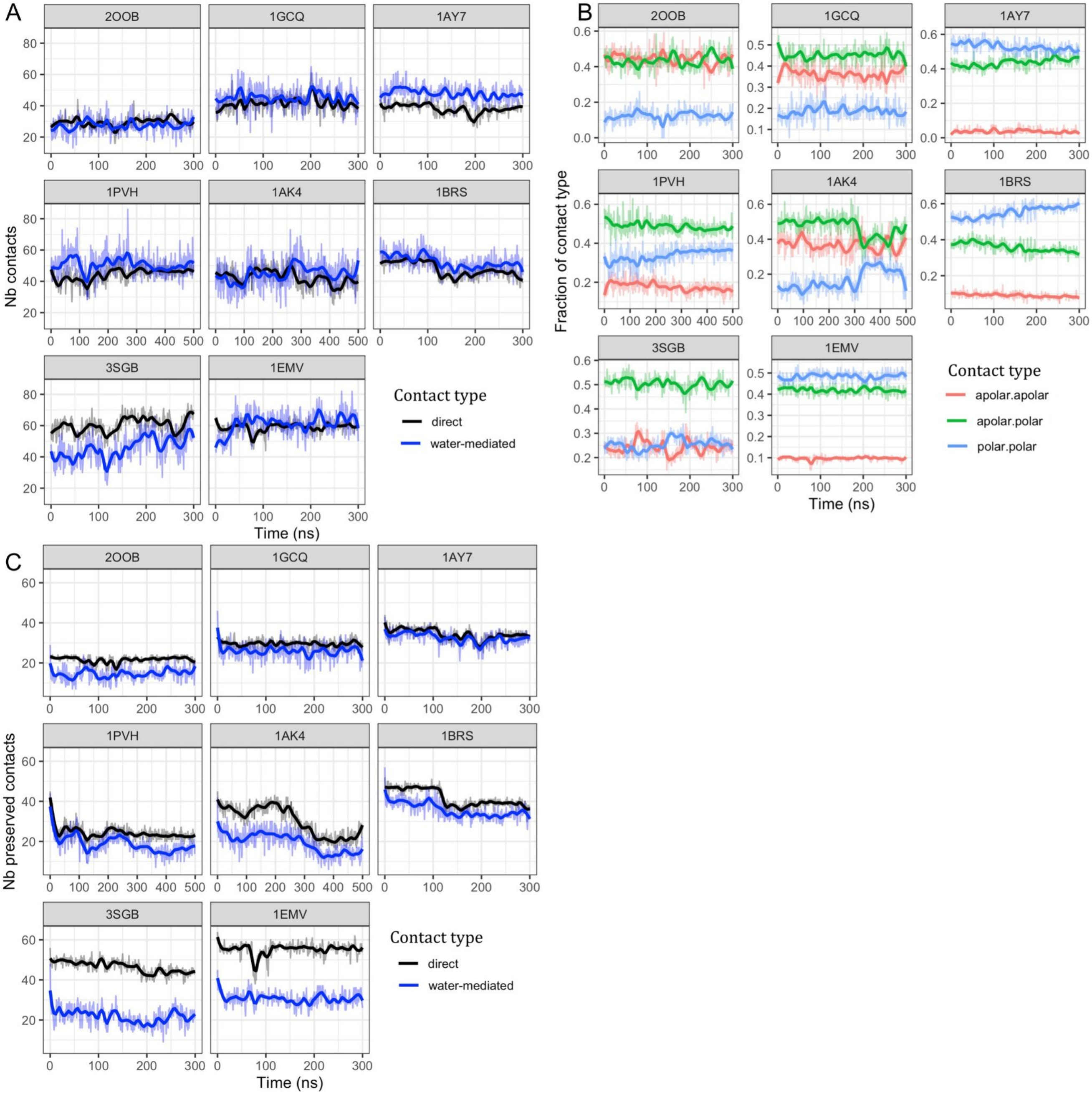

Supplementary Figure 8. Number of interface contacts. A: time series of the number of interface contacts, direct or mediated by water molecules, B: time series of the fraction of each contact type at the interface (polar residues : ASP, GLU, ASN, GLN, SER, THR, TYR, LYS, ARG, HIS, apolar residues: ALA, VAL, LEU, ILE, PRO, PHE, MET, TRP, GLY ,CYS), C: time series of the number of initial contacts that are preserved, direct or mediated by water molecules.

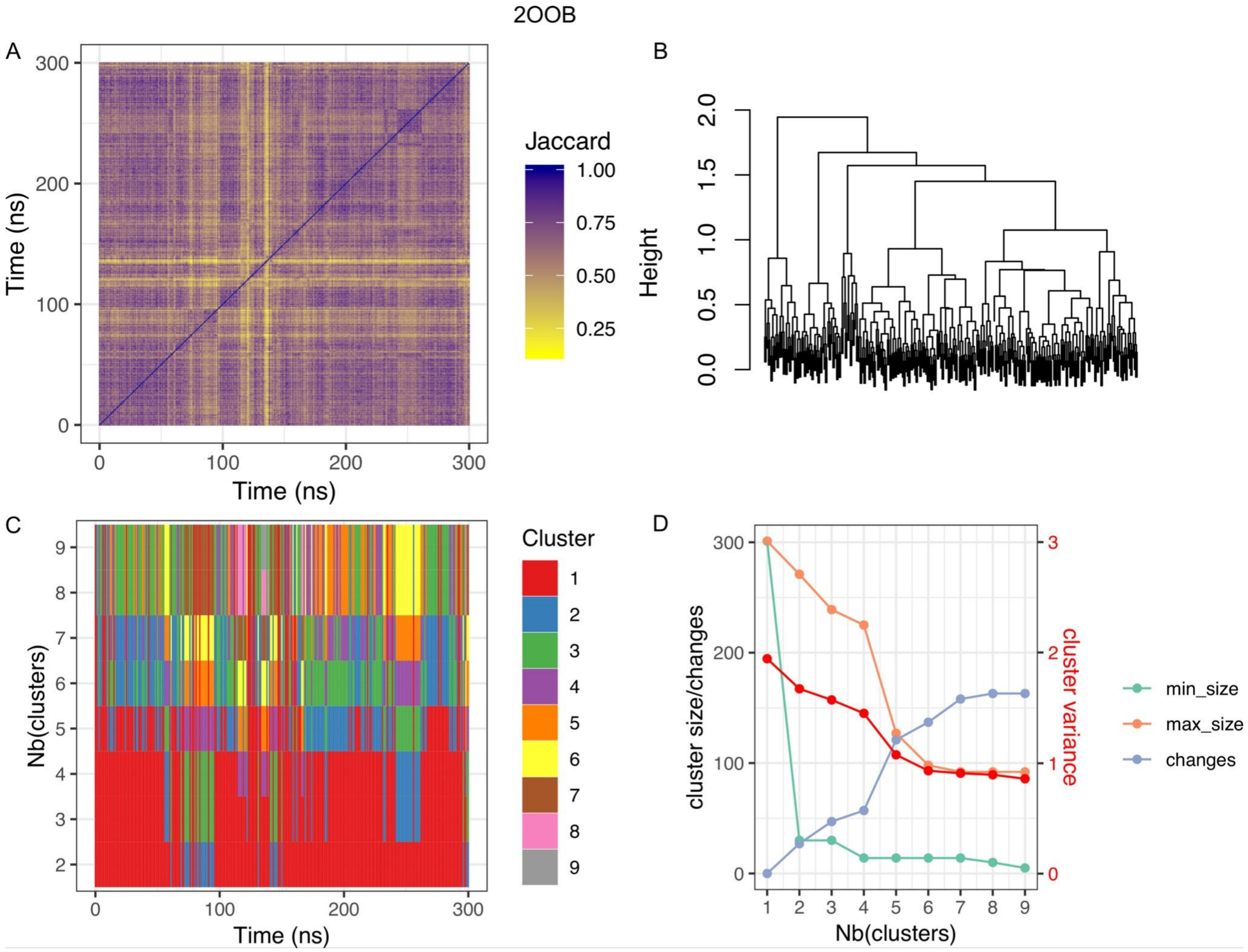

Supplementary Figure 9. Clustering results for complex 2OOB (1 cluster). A: Jaccard similarity matrix; for each pair of snapshots, a yellow pixel indicates low interface similarity and a purple pixel indicates high interface similarity. B: clustering dendrogram. C: cluster membership along simulation time, for different numbers of clusters. D: cluster size, number of changes, and intra-cluster variance (red), for different numbers of clusters; min_size: size of the smallest cluster, max_size: size of the largest cluster, changes: number of cluster changes during the simulation.

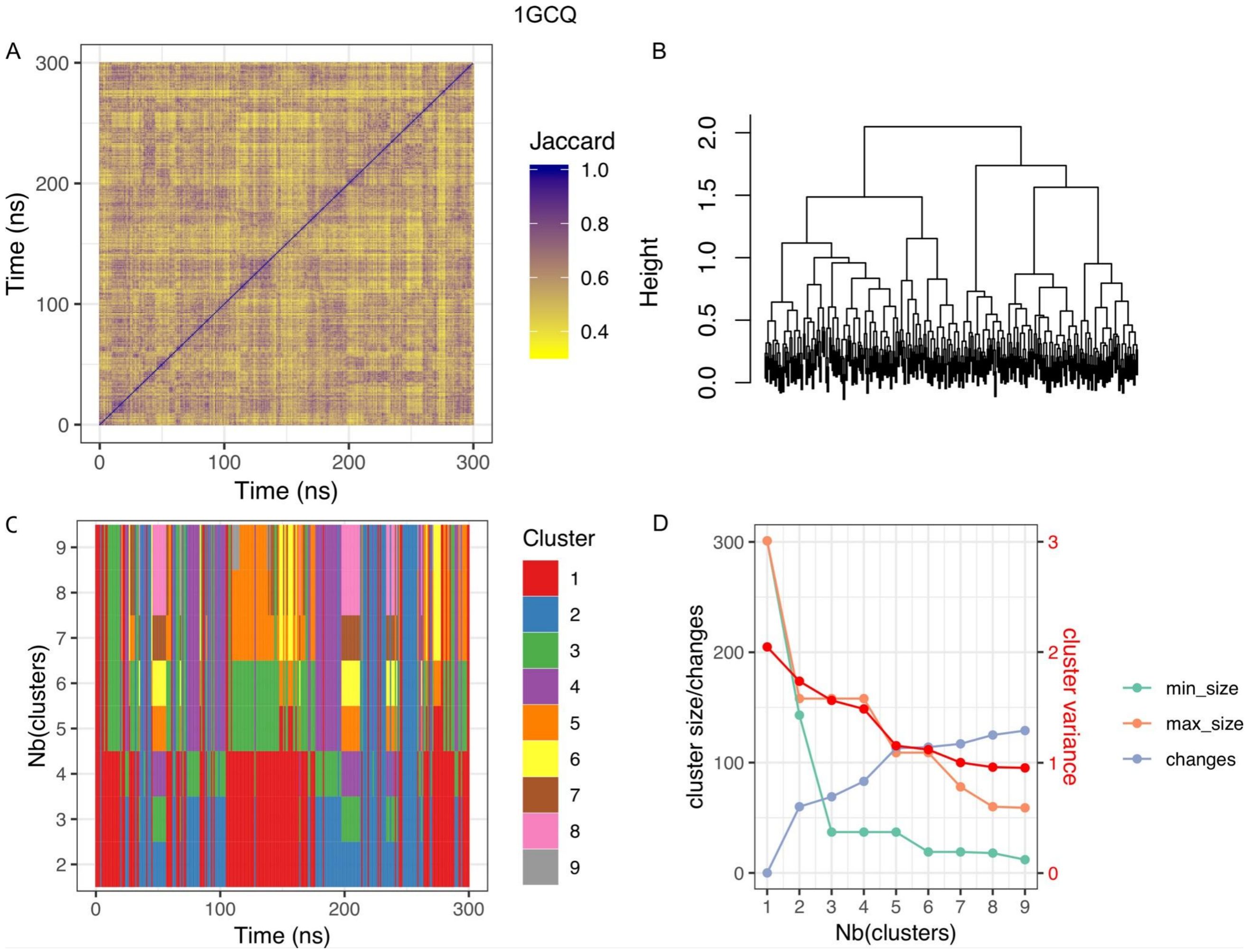

Supplementary Figure 10. Clustering results for complex 1GCQ (1 cluster). See legend of Figure S9 for details.

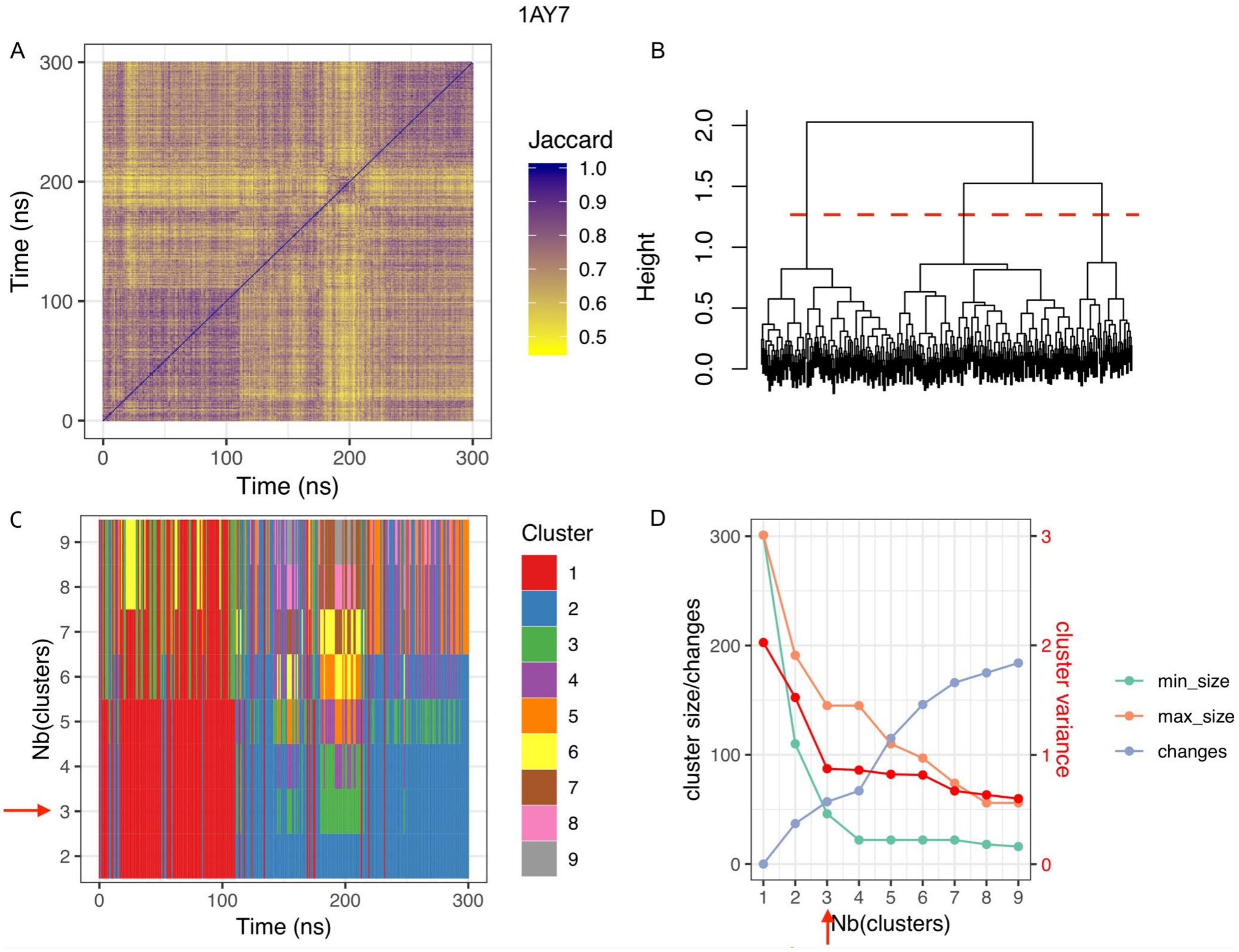

Supplementary Figure 11. Clustering results for complex 1AY7 (3 clusters). See legend of Figure S7 for details. The red dashed line in panel B and the red arrows in panels C and D indicate the optimal number of clusters.

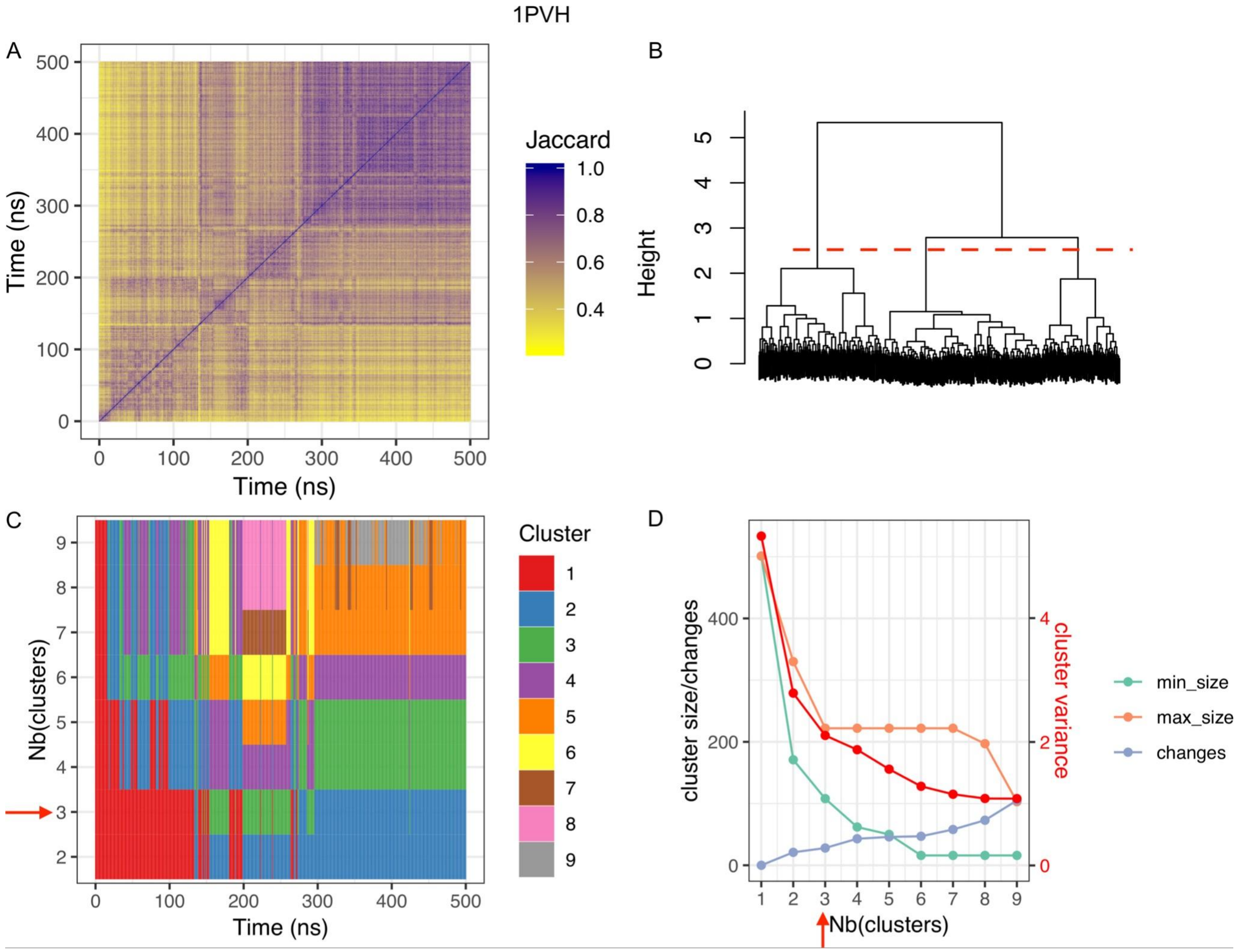

Supplementary Figure 12. Clustering results for complex 1PVH (3 clusters). See legend of Figure S9 for details. The red dashed line in panel B and the red arrows in panels C and D indicate the optimal number of clusters.

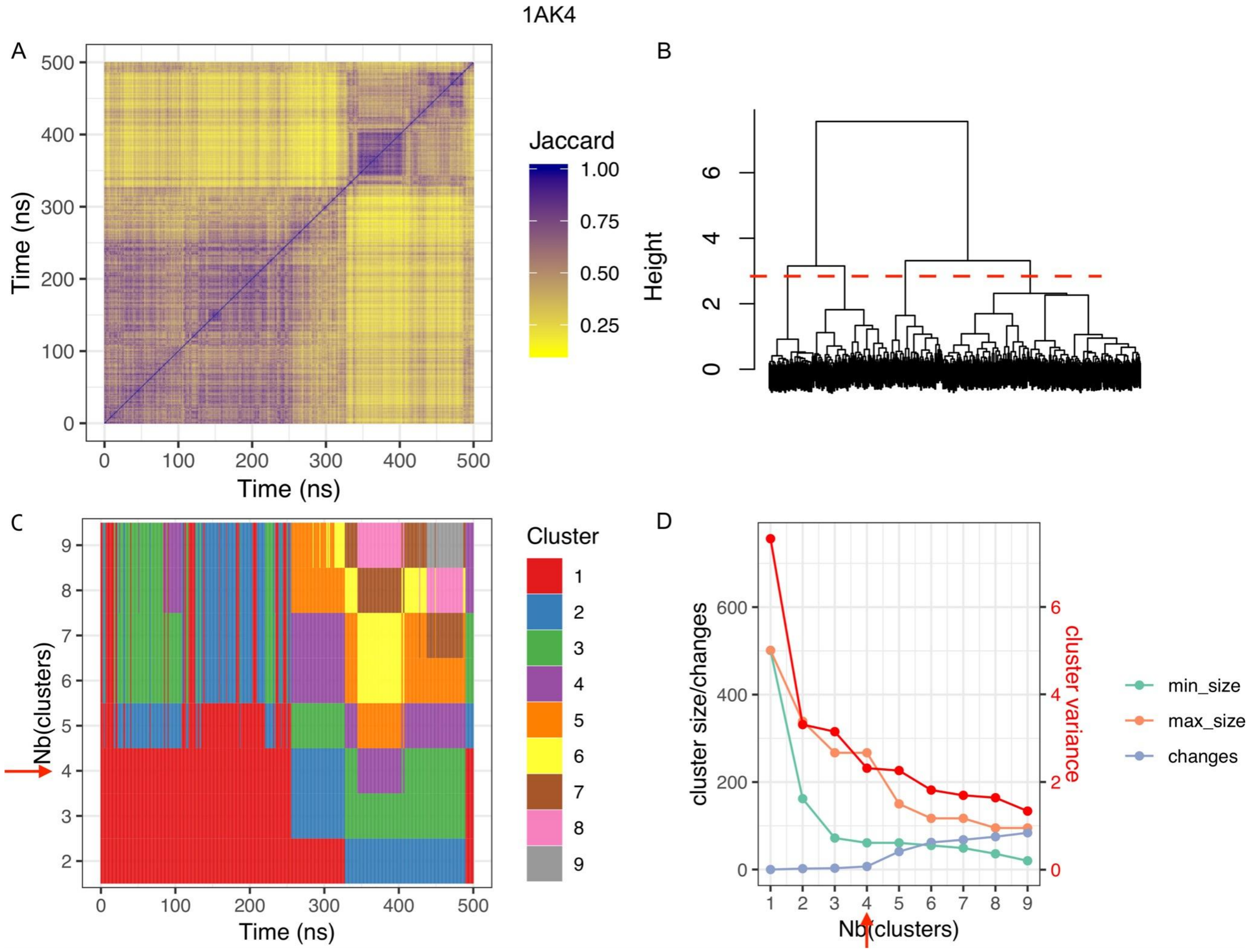

Supplementary Figure 13. Clustering results for complex 1AK4 (4 clusters). See legend of Figure S7 for details. The red dashed line in panel B and the red arrows in panels C and D indicate the optimal number of clusters.

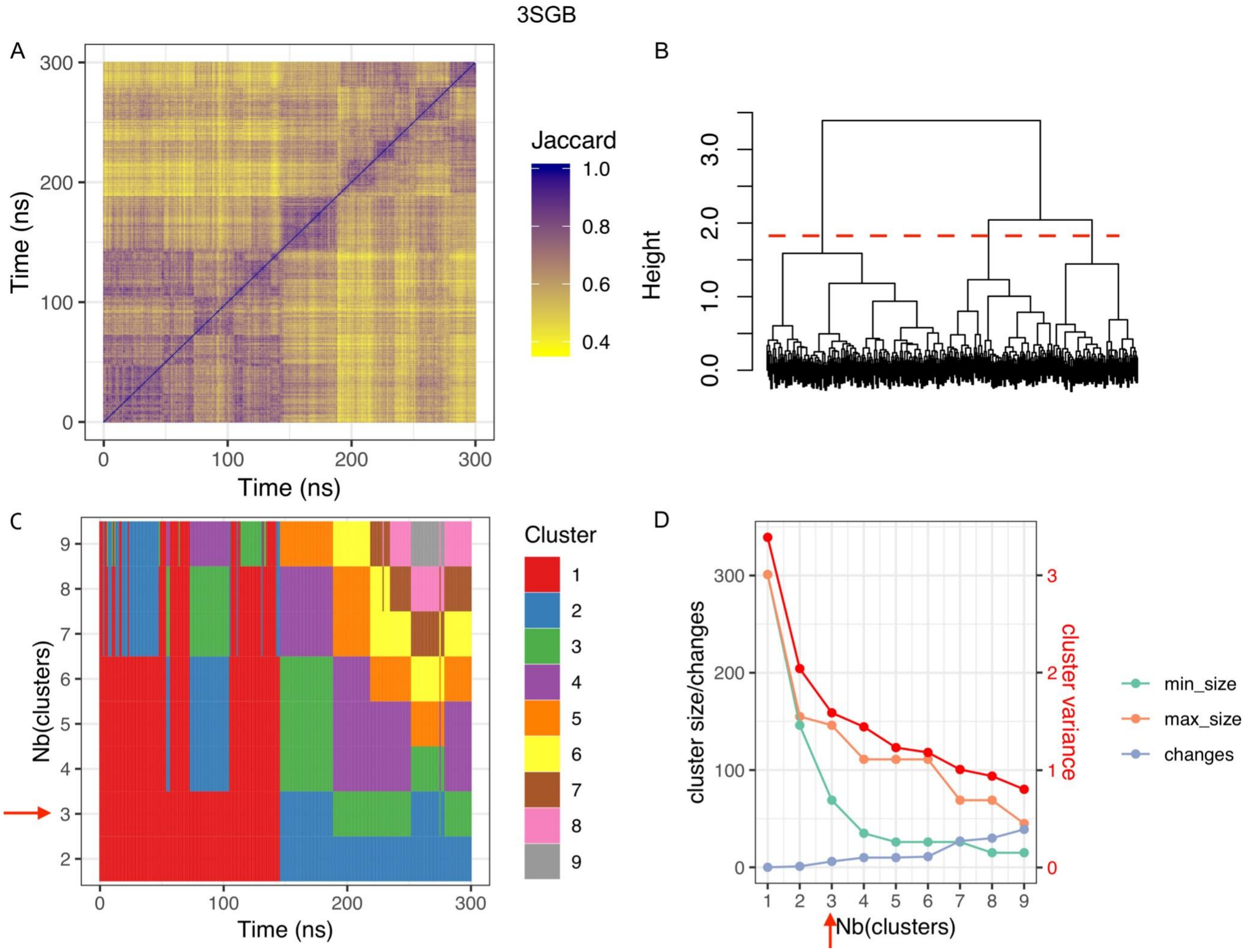

Supplementary Figure 14. Clustering results for complex 3SGB (3 clusters). See legend of Figure S9 for details. The red dashed line in panel B and the red arrows in panels C and D indicate the optimal number of clusters.

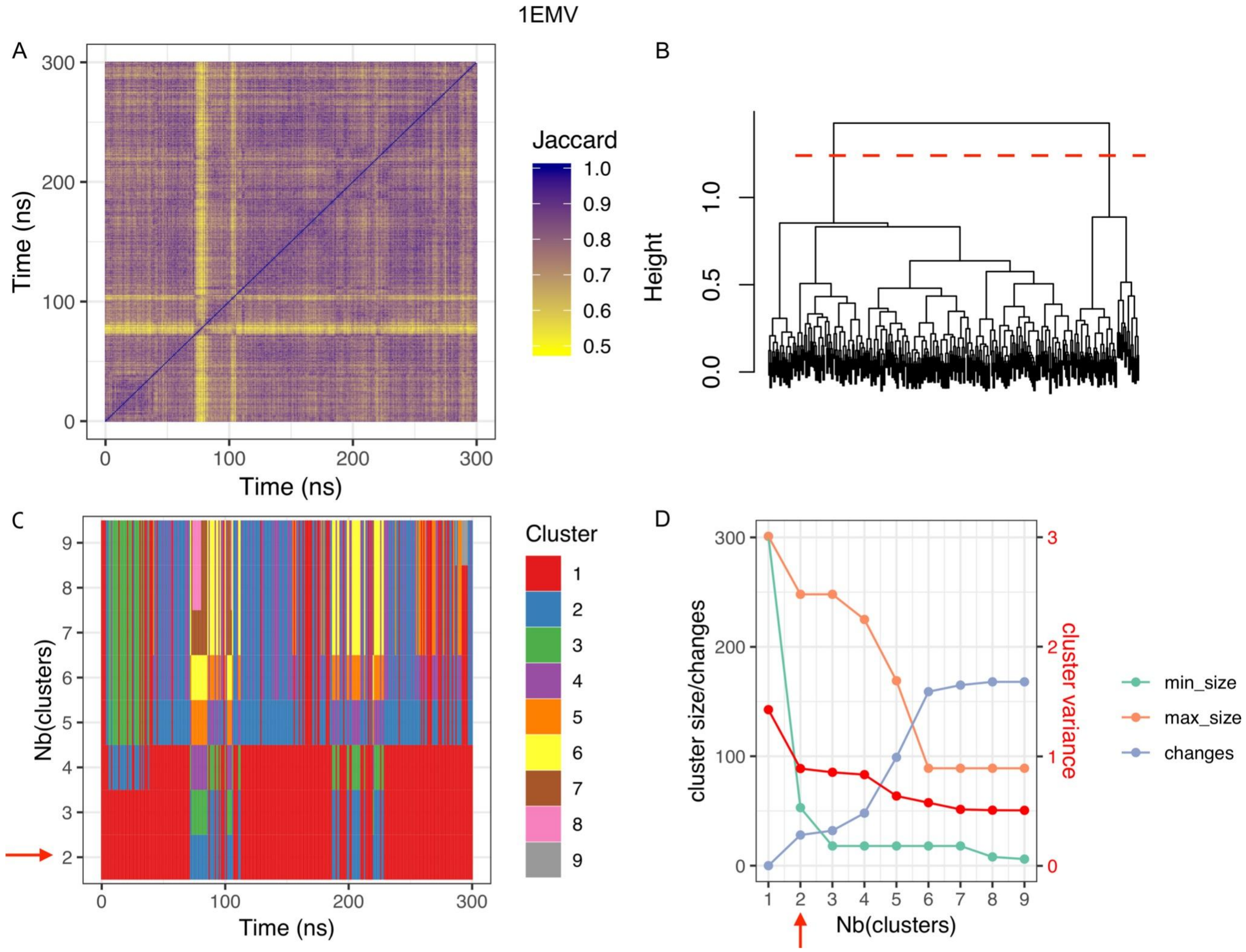

Supplementary Figure 15. Clustering results for complex 1EMV (2 clusters). See legend of Figure S7 for details. The red dashed line in panel B and the red arrows in panels C and D indicate the optimal number of clusters.

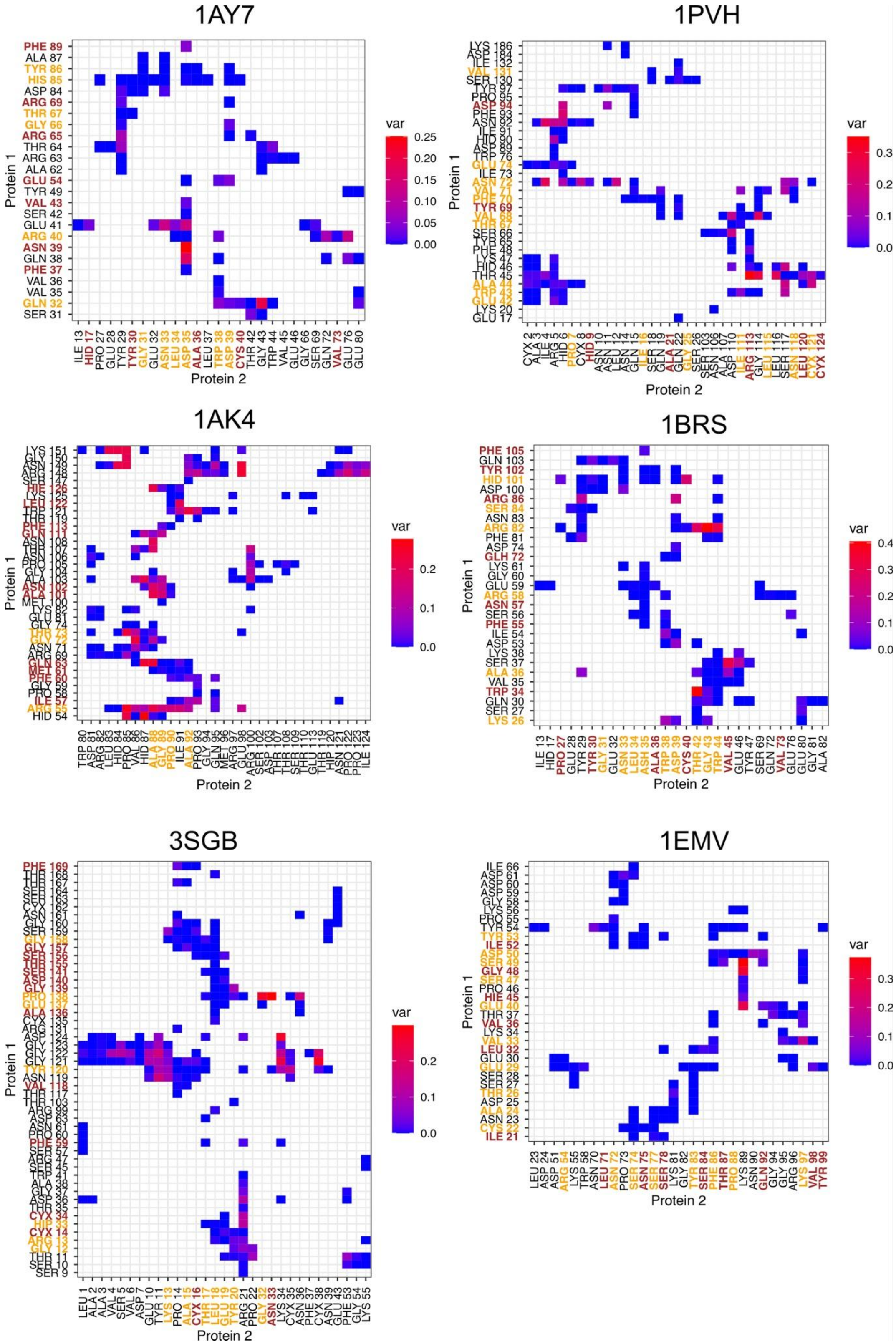

Supplementary Figure 16. Variance of contact frequencies across clusters. Residues labeled in orange are classified as core, and residues in brown as support in the experimental structure.

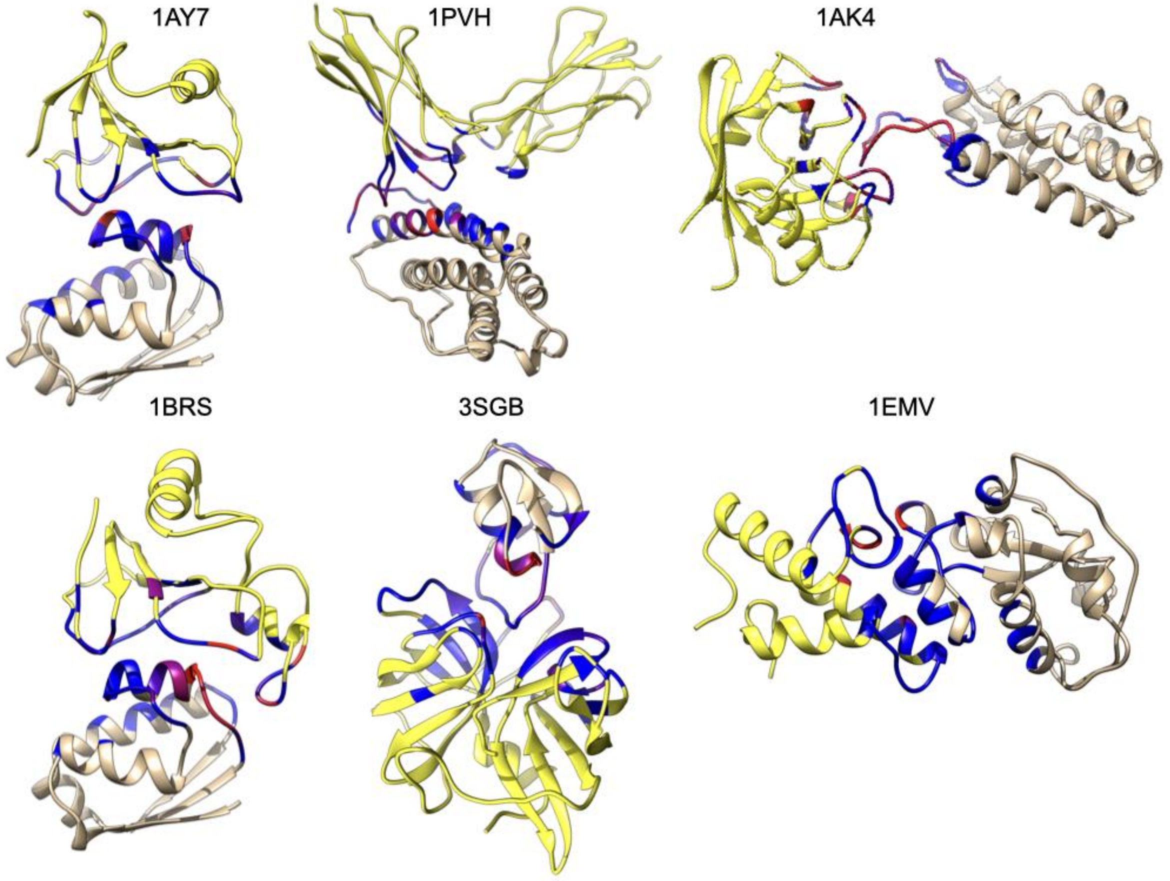

Supplementary Figure 17. 3D structures colored by contact variance, blue= low variance, red=high variance.

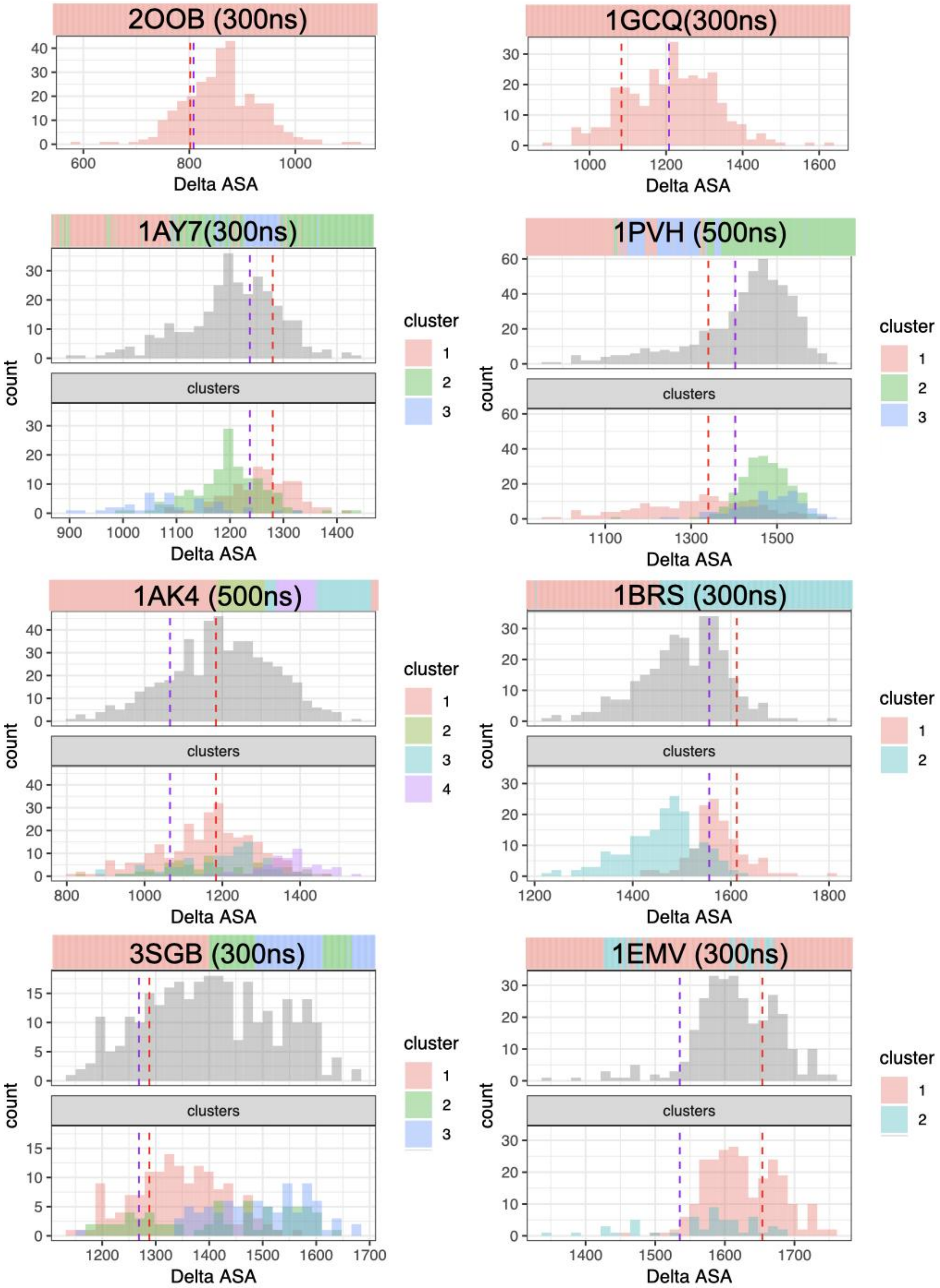

Supplementary Figure 18. Distribution of interface ΔASA (Å^2). For each complex, the global distribution is plotted in gray and the distributions in each cluster in colors. The colored ribbon at the top of each plot represents the time series of visited clusters during the simulation. Red dotted lines indicate initial values and purple dotted lines indicate values in the crystal structures.

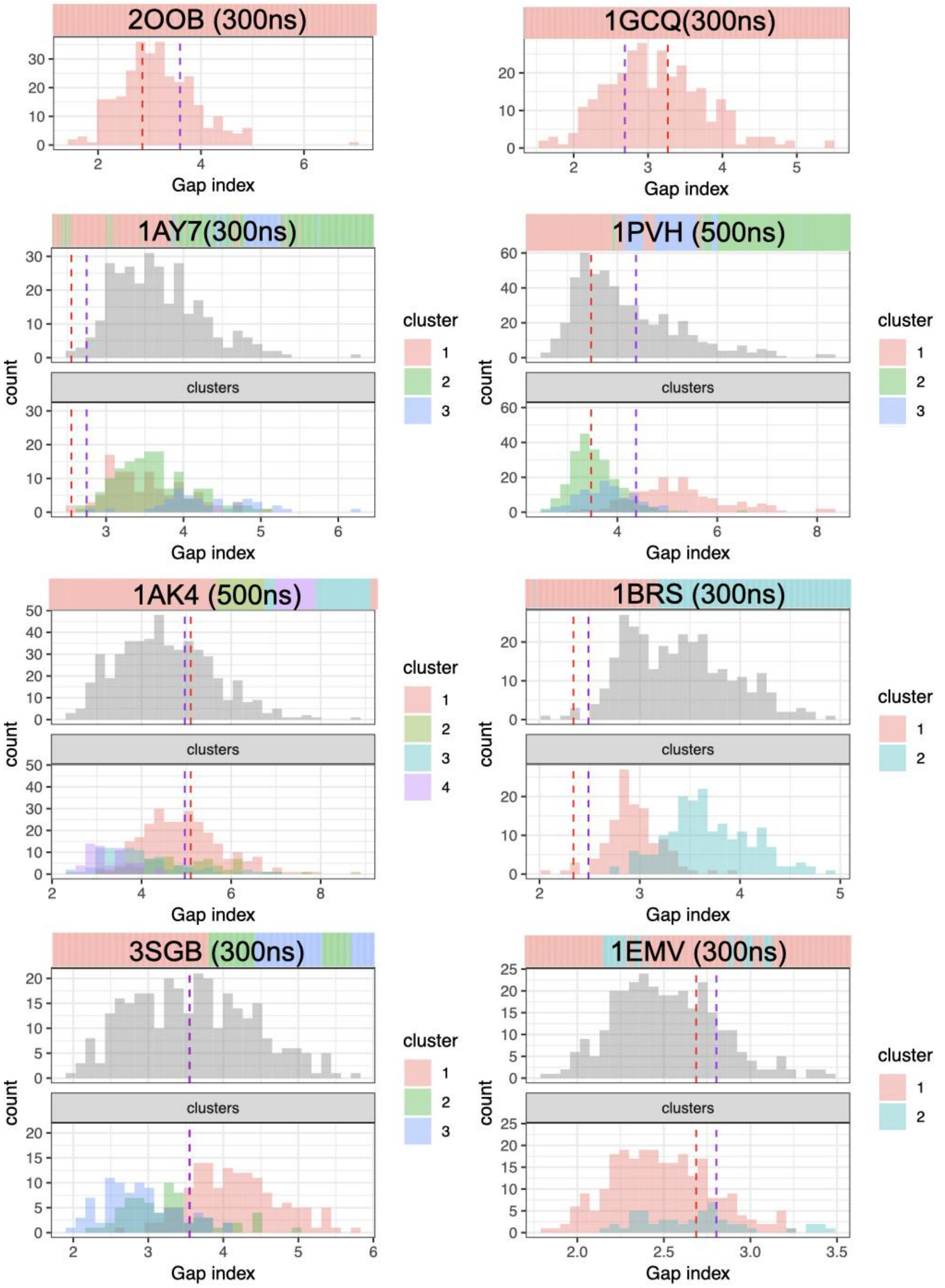

Supplementary Figure 19. Distribution of the gap index (Å) in each sub-state for each complex. See Figure S18 for details.

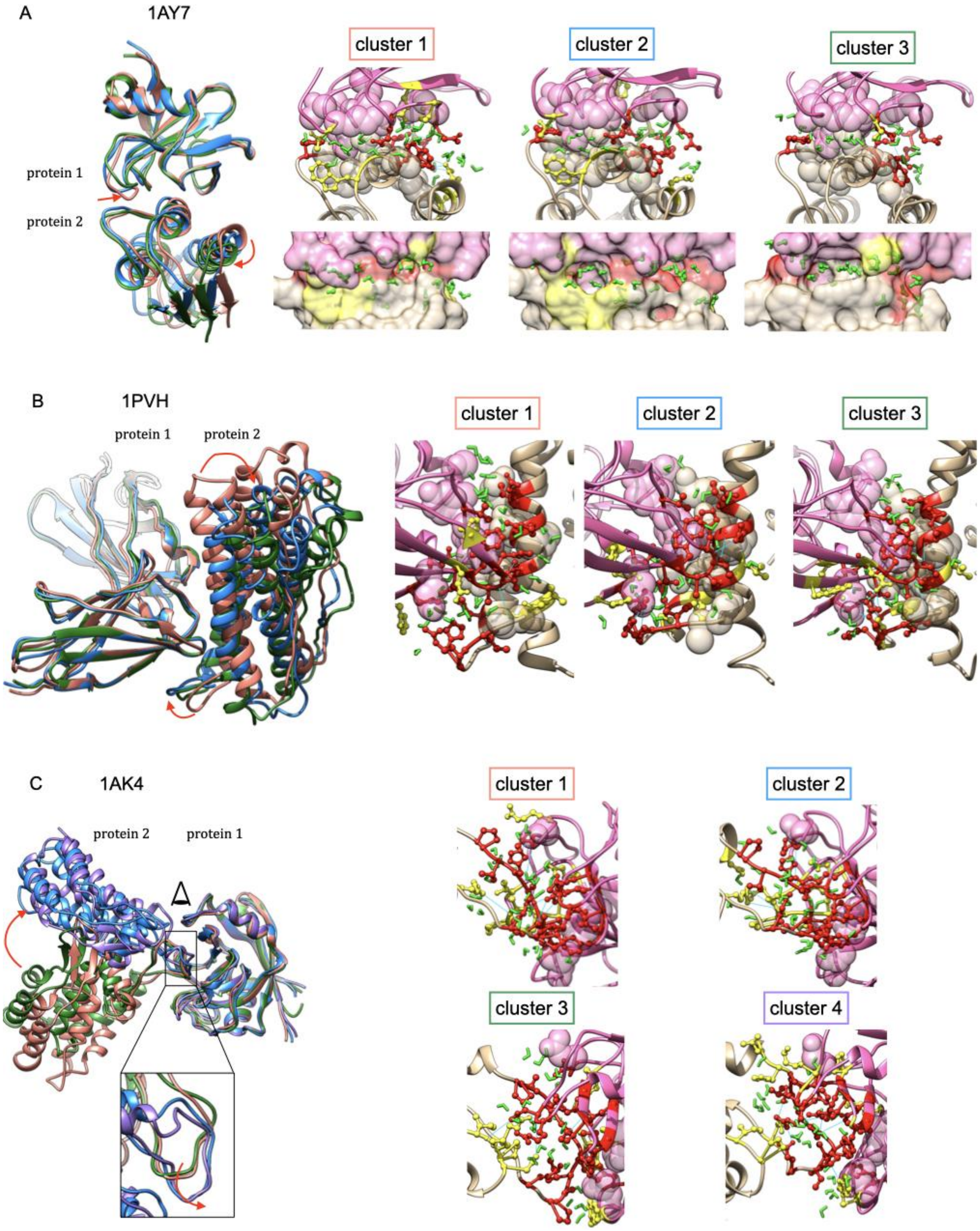

Supplementary Figure 20. Representative structures of interface clusters for complexes 1AY7, 1PVH and 1AK4. Left part: global superimposition of structure representatives. Interfaces in each cluster are represented with one protein in pink and the other in beige, with water molecules in green. Interface residues are represented as follows: residues involved in stable contacts as spheres, residues involved in variable contacts as red balls and sticks, residues involved in contacts that are specific to one cluster in yellow balls and sticks.

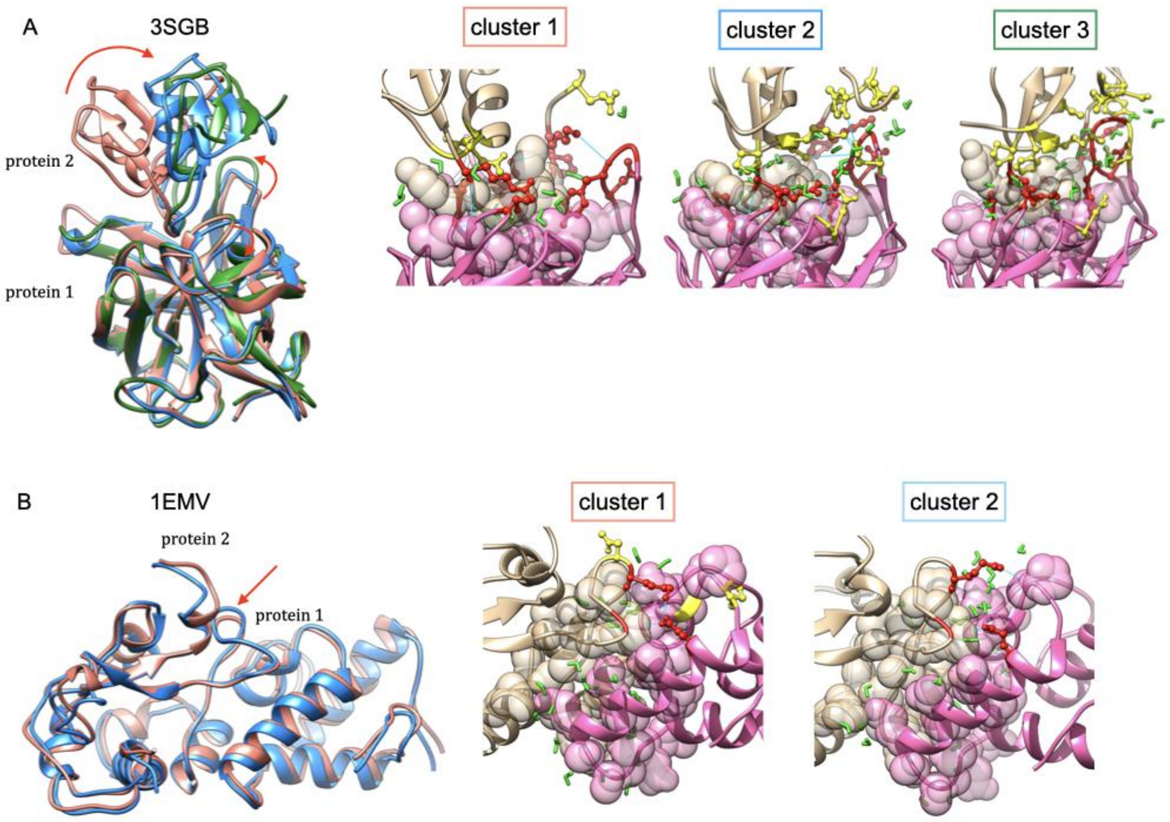

Supplementary Figure 21. Representative structures of interface clusters for complexes 3SGB and 1EMV. See Figure S20 for details.

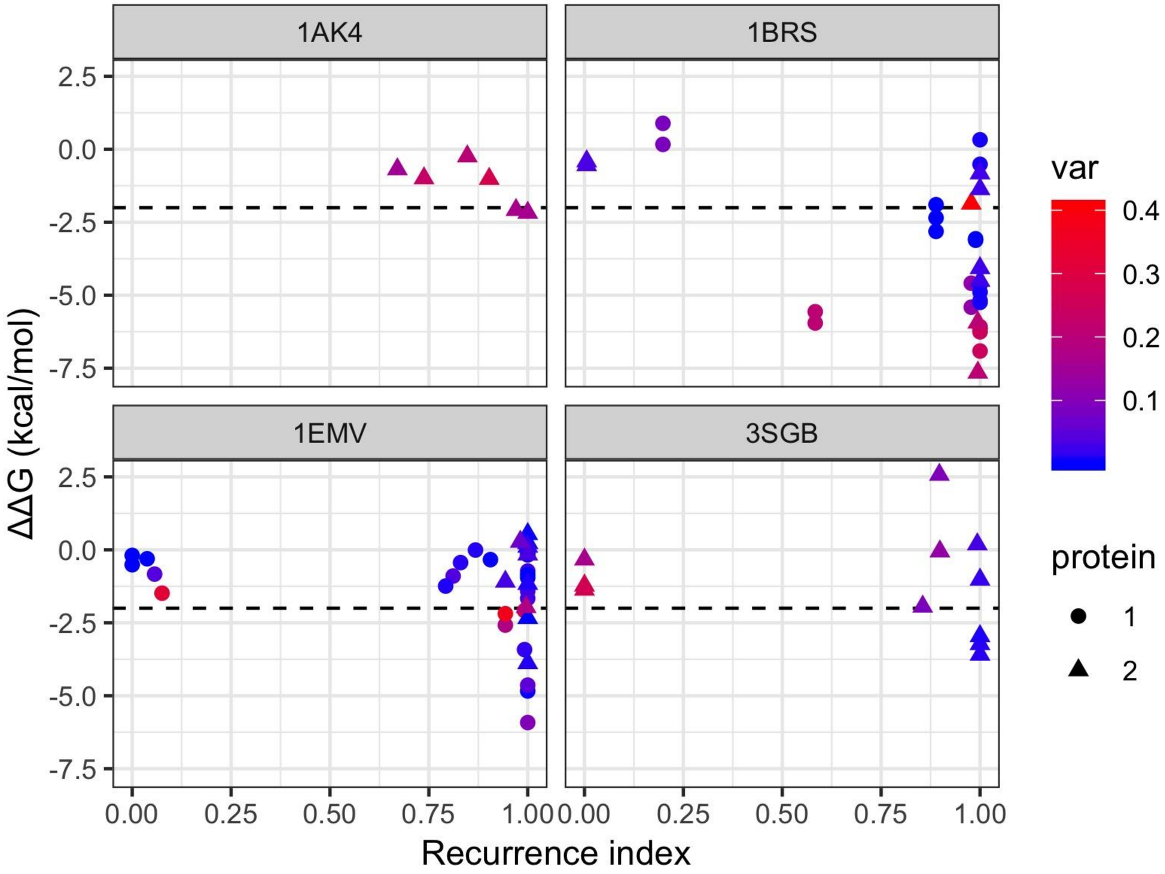

Supplementary Figure 22. Comparison of ΔΔG values upon mutation from SKEMPIv2.0 with recurrence index and contact variance mapped for each residue.

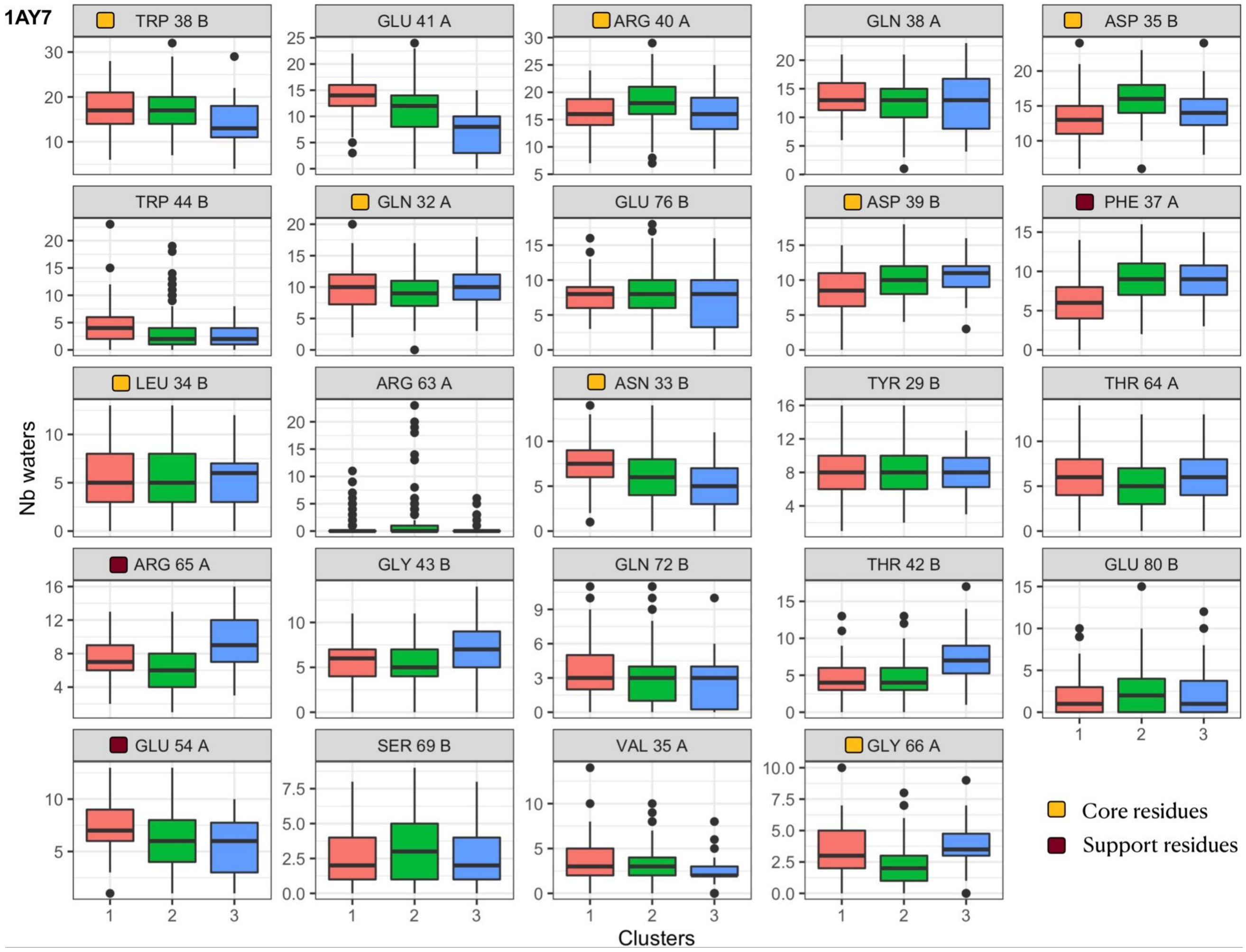

Supplementary Figure 23. Number of interface water molecules in contact with each interface residue in the ribonuclease/barstar complex 1AY7. Distributions are shown as boxplots in each interface cluster. Orange squares indicate core residues and brown squares indicate support residues in the experimental structure. Hot spot information not available.

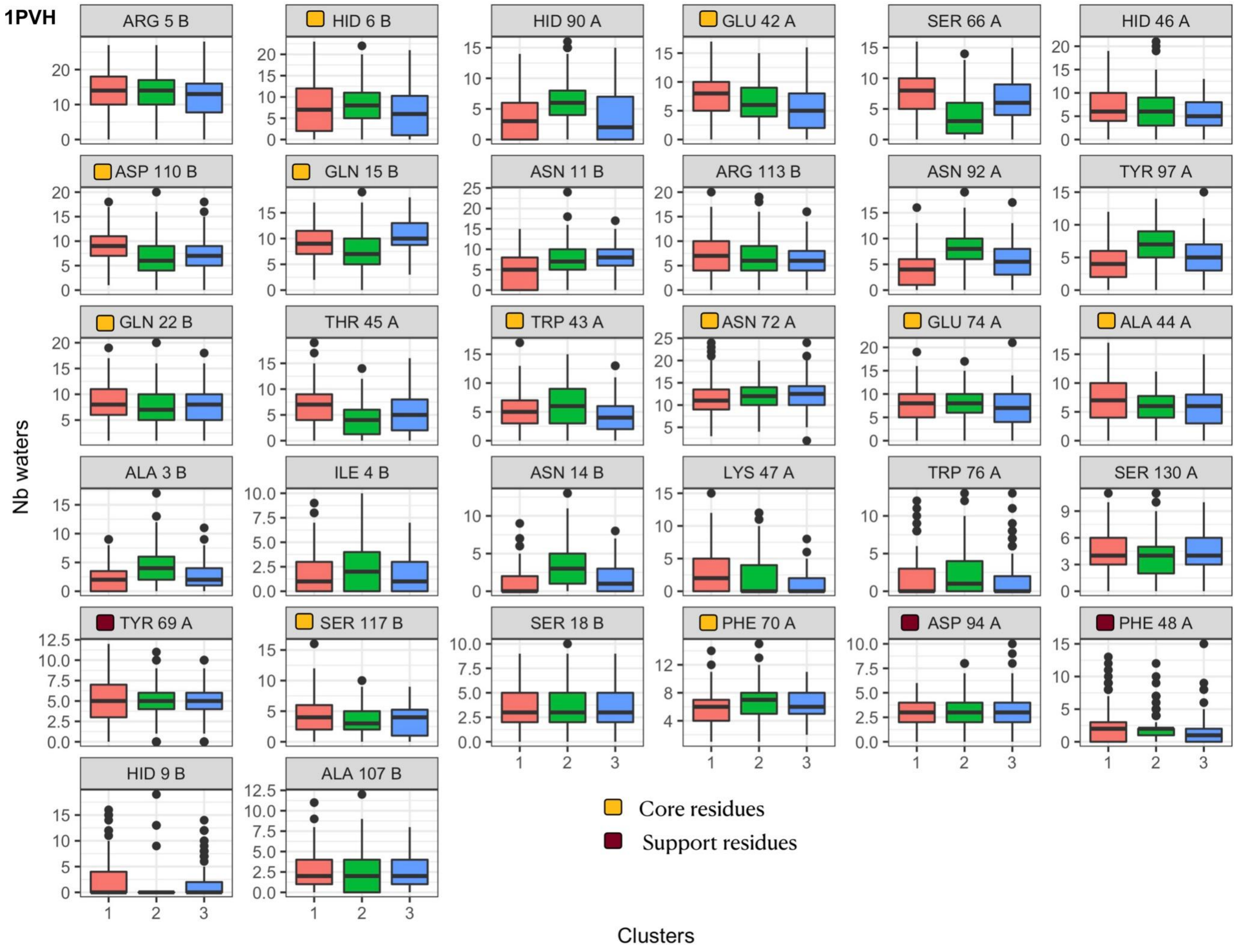

Supplementary Figure 24. Number of interface water molecules in contact with each interface residue in the Interleukine 6 receptor/leukemia inhibitory factor complex 1PVH. See Figure S23 for details. Hot spot information not available.

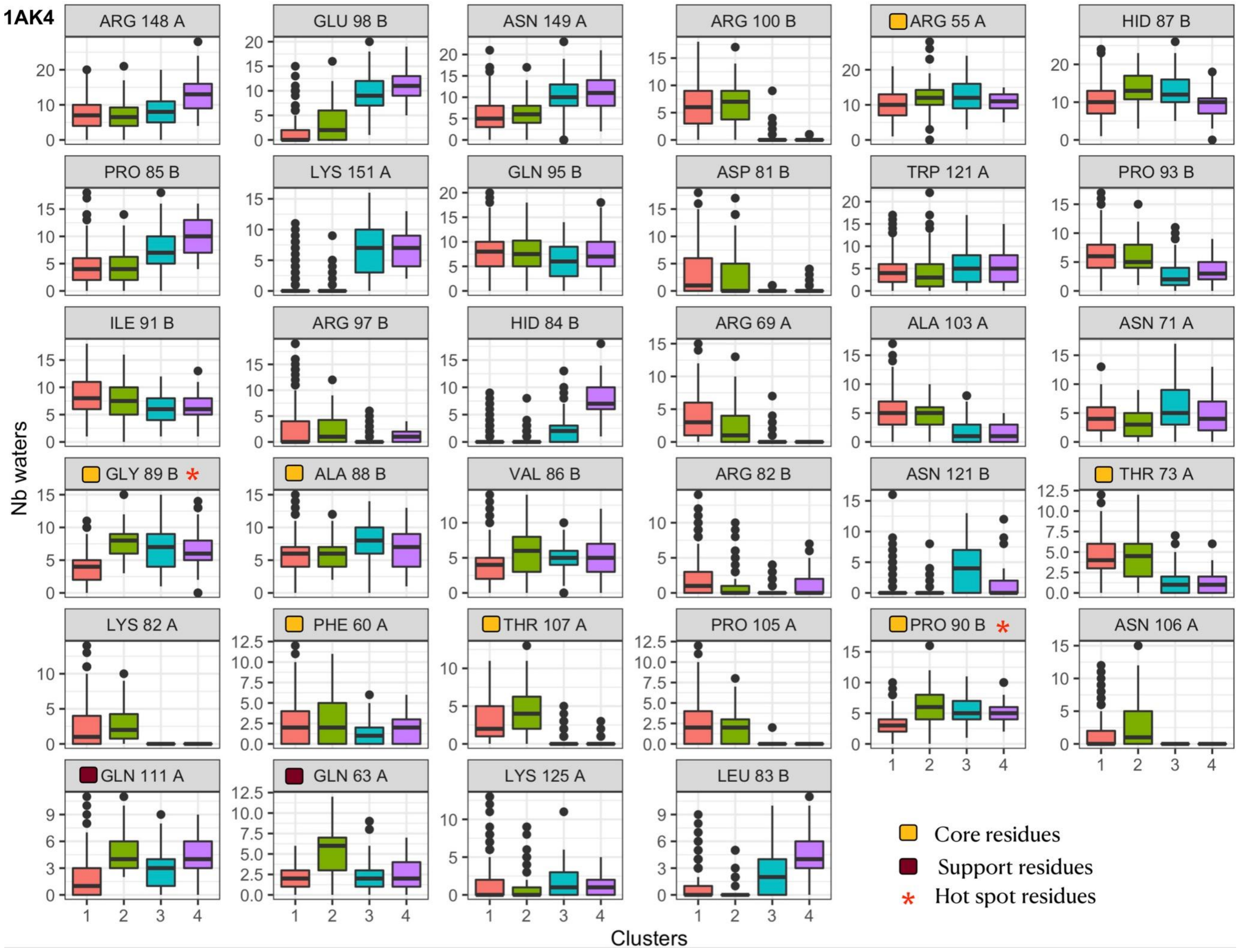

Supplementary Figure 25. Number of interface water molecules in contact with each interface residue in the cyclophilin/HIV capsid complex 1AK4. See Figure S23 for details. Red stars indicate hot spot residues. Hot spot information is available only for the second protein.

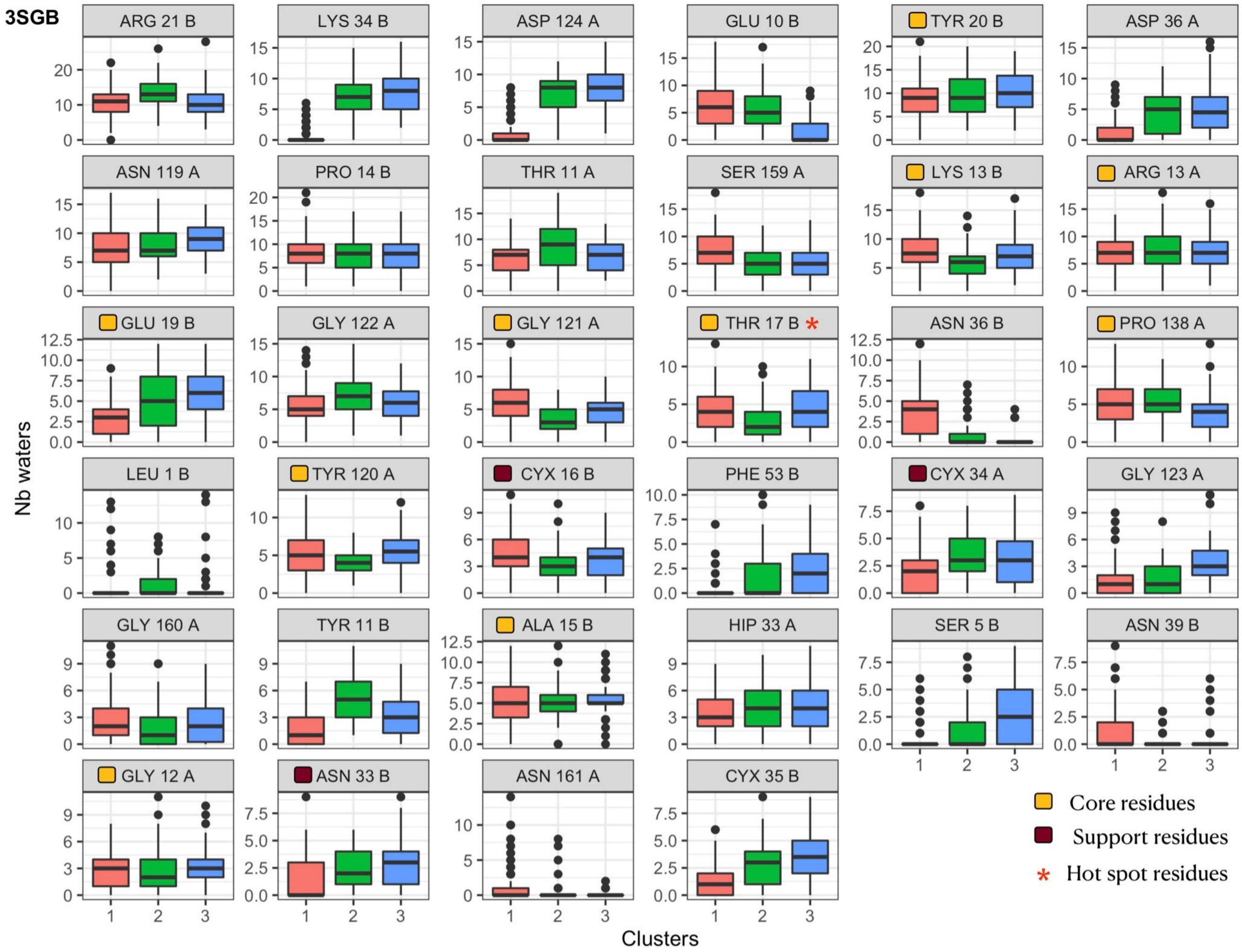

Supplementary Figure 26. Number of interface water molecules in contact with each interface residue in the streptogrisin/inhibitor complex 3SGB. See Figure S23 for details. Red stars indicate hot spot residues. Hot spot information is available only for the second protein.

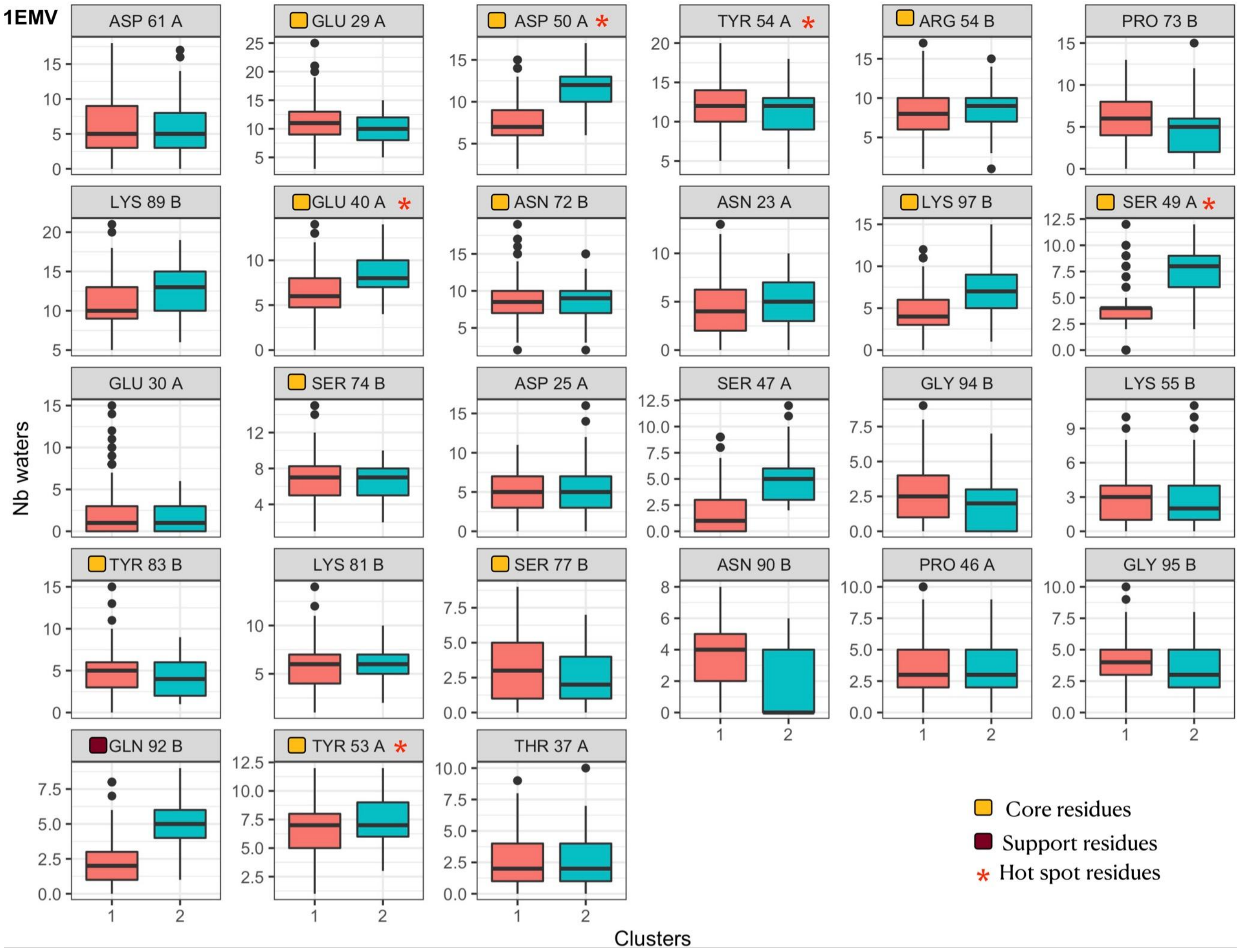

Supplementary Figure 27. Number of interface water molecules in contact with each interface residue in the colicin endonuclease/inhibitor complex 1EMV. See Figure S23 for details. Red stars indicate hot spot residues.

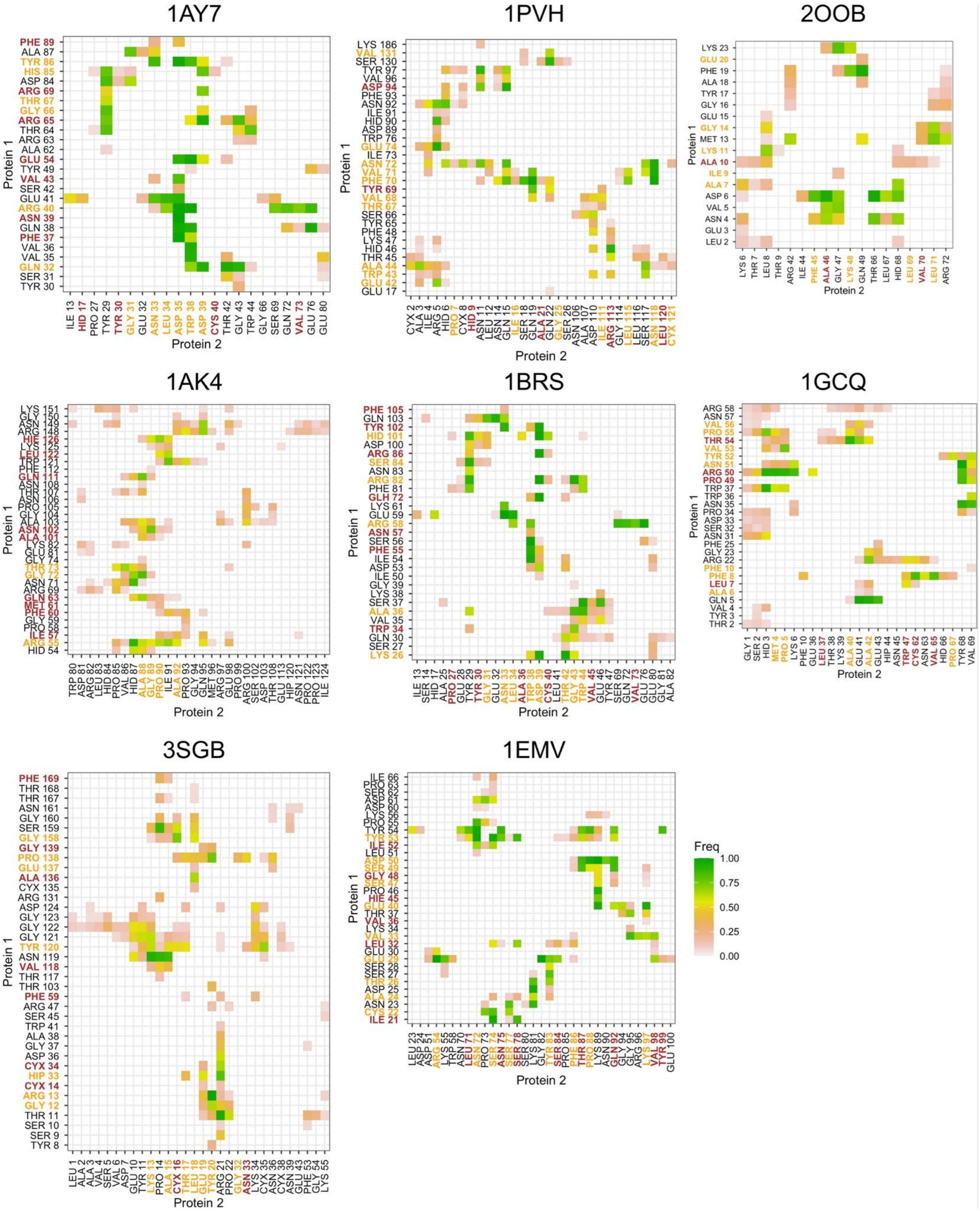

Supplementary Figure 28. Relative frequency of water-mediated contacts in all the complexes. Residues labeled in orange are classified as core and residues in brown as support in the experimental structures.
